# DPPA2/4 and SUMO E3 ligase PIAS4 opposingly regulate zygotic transcriptional program

**DOI:** 10.1371/journal.pbio.3000324

**Published:** 2019-06-21

**Authors:** Yao-Long Yan, Chao Zhang, Jing Hao, Xue-Lian Wang, Jia Ming, Li Mi, Jie Na, Xinli Hu, Yangming Wang

**Affiliations:** 1 Institute of Molecular Medicine, Peking University, Beijing, China; 2 Academy for Advanced Interdisciplinary Studies, Peking University, Beijing, China; 3 School of Medicine, Tsinghua University, Beijing, China; Icahn School of Medicine at Mount Sinai, UNITED STATES

## Abstract

The molecular mechanism controlling the zygotic genome activation (ZGA) in mammals remains poorly understood. The 2-cell (2C)-like cells spontaneously emerging from cultures of mouse embryonic stem cells (ESCs) share some key transcriptional and epigenetic programs with 2C-stage embryos. By studying the transition of ESCs into 2C-like cells, we identified developmental pluripotency associated 2 and 4 (Dppa2/4) as important regulators controlling zygotic transcriptional program through directly up-regulating the expression of double homeobox (Dux). In addition, we found that DPPA2 protein is sumoylated and its activity is negatively regulated by small ubiquitin-like modifier (Sumo) E3 ligase protein inhibitor of activated STAT 4 (PIAS4). PIAS4 is down-regulated during ZGA process and during transitioning of ESCs into 2C-like cells. Depleting Pias4 or overexpressing Dppa2/4 is sufficient to activate 2C-like transcriptional program, whereas depleting Dppa2/4 or forced expression of Pias4 or Sumo2–Dppa2 inhibits 2C-like transcriptional program. Furthermore, ectopic expression of Pias4 or Sumo2–Dppa2 impairs early mouse embryo development. In summary, our study identifies key molecular rivals consisting of transcription factors and a Sumo2 E3 ligase that regulate zygotic transcriptional program upstream of Dux.

## Abstract 2

Analysis of cultured cells resembling those from 2-cell-stage embryos identifies transcription factors Dppa2/4 and the Sumo2 E3 ligase PIAS4 as key antagonistic regulators of the zygotic transcriptional program upstream of Dux.

## Introduction

Zygotic genome activation (ZGA) occurs predominantly at the 2-cell (2C) stage of mouse embryo and 4- to 8-cell stages in human embryo [1–3], which is essential for the development control passed from maternal to newly synthesized RNA and proteins. Any wrongdoing during ZGA may lead to termination of embryo development or have severe and long-term consequences for the life of an organism. However, the molecular regulation of ZGA in mammal is poorly understood. Recently, a rare subset of cells called 2C-like cells were found in mouse embryonic stem cell (ESC) cultures [4,5]. The 2C-like cells express high levels of ZGA transcripts including murine endogenous retrovirus (ERV)-L (MERVL) family of retroviruses and zinc finger and SCAN domain containing 4 (Zscan4) clusters [5–7]. In addition, these cells share some key epigenetic characteristics with 2C-stage embryos, including increased chromatin accessibility [6], the absence of chromocenters [8], and high histone mobility [9]. Therefore, 2C-like cells serve as an ideal cell model system to study the regulation of ZGA transcriptional program.

Recently, the transcription factor double homeobox (DUX), expressed exclusively in the minor wave of ZGA, was reported to activate numerous downstream ZGA transcripts [10–12]. Deletion of Dux causes severe defects in preimplantation development. Consistently, ectopic expression of Dux is sufficient to promote the transition of ESCs to 2C-like cells in mouse. In addition, several studies show that the 2C-like transcriptional program can be activated by depleting microRNA 34a (miR-34a) [13], chromatin assembly factor (CAF-1) [8], various epigenetic regulators including lysine (K)-specific demethylase 1A (Lsd1/Kdm1a) [5], components PRC1.6 complex and E1A binding protein p400 (EP400)–TIP60 complex [7], and long interspersed nuclear element (LINE1)–Nucleolin complex [14]. In particular, tripartite motif-containing 28 (Trim28), PRC1.6 complex, and LINE1–Nucleolin complex directly bind to Dux genomic loci to repress its expression [7,14]. These reported factors are generally repressors of Dux or ZGA transcripts. Furthermore, two recent studies found that transcription factors developmental pluripotency associated 2 (Dppa2) and Dppa4 are both necessary and sufficient for the activation of Dux and other 2C genes [15,16].

A recent study identified small ubiquitin-like modifier 2 (SUMO2) as a potent inhibitor of provirus and ERVs including MERVL [17], indicating a potential role of sumoylation in regulating 2C-like state and zygotic transcriptional program. Posttranslational modification by SUMO is an important regulatory mechanism to tune protein function [18,19]. Numerous transcriptional factors [20,21] and chromatin modifiers [22–24] are shown to be regulated by sumoylation. Sumoylation also plays vital roles in the transcriptional response to various intrinsic or extrinsic stresses, including DNA damage [25,26], oxidative stress [27,28], heat shock [29], and pathogen invasion [30]. However, whether and how sumoylation regulates zygotic transcriptional program remains unknown. In this study, we set out to determine the function of sumoylation factors in regulating 2C-like state and zygotic transcriptional program. We identified a Sumo2 E3 ligase protein inhibitor of activated STAT 4 (Pias4) and its substrate Dppa2 that play opposing roles in the regulation of 2C-like state and zygotic transcriptional program upstream of Dux. In addition, Dppa4 functions together with Dppa2 to activate the zygotic transcriptional program.

## Results

### Sumo2 and Sumo E3 ligase Pias4 inhibit zygotic transcriptional program in mouse ESCs

To study how zygotic transcriptional program is regulated, we first generated a 2C::tandem dimeric Tomato (tdTomato) reporter ESC line with transgenic expression of red fluorescence protein tdTomato under the control of MERVL 5′UTR [5]. As expected, around 0.5% 2C::tdTomato-positive cells were typically present in ESC cultures (S1A Fig). Using this cell line, we confirmed that knocking down Sumo2, but not Sumo1, significantly increased the percentage of tdTomato-positive cells (S1A Fig). Importantly, knocking down Sumo2 also up-regulated the expression of MERVL and other classic 2C-specific genes including Dux, Cml2, zinc finger protein 352 (Zfp352), and Zscan4d (S1B Fig).

**Fig 1:**
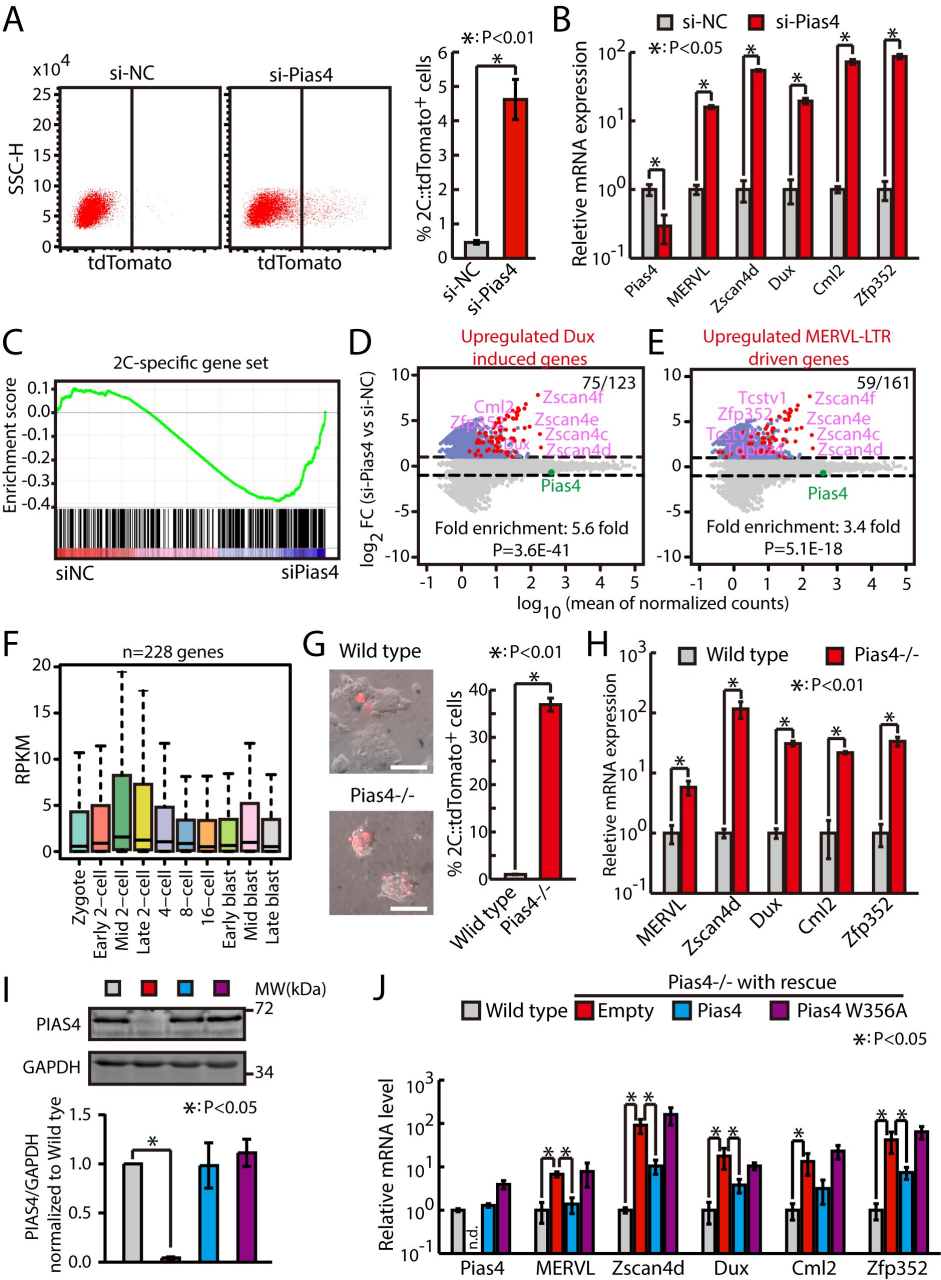
Pias4 represses zygotic transcriptional program in mouse ESCs. (A) Flow cytometry analysis of 2C::tdTomato-positive cells in Pias4-knockdown ESCs. Left, representative dot plot; right, quantification of fraction of tdTomato-positive cells in 2C::tdTomato ESCs treated with control siRNAs or siRNAs against Pias4. Shown are mean ± SD, *n* = 6. The *p*-value was calculated by two-tailed Student’s *t* test. (B) RT-qPCR of Dux and other 2C-specific genes in Pias4-knockdown ESCs. The β-actin gene was used as a control. For each gene, data were normalized to the mRNA level of ESCs transfected with control siRNAs. Shown are mean ± SD, *n* = 3. The *p*-value was calculated by two-tailed Student’s *t* test. (C) GSEA for 2C-specific genes in ESCs transfected with control siRNAs or siRNAs against Pias4. For x-axis, genes were ranked based on the ratio of siNC versus si-Pias4 ESCs. (D) MA plots showing gene expression changes in Pias4-knockdown ESCs. Red dots indicate Dux-induced genes. Out of 123 Dux-induced genes, 75 were up-regulated in Pias4-knockdown ESCs. Fold enrichment and *p*-value are shown. The *p*-value was calculated by hypergeometric test. (E) MA plots showing gene expression changes in Pias4-knockdown ESCs. Red dots indicate MERVL–LTR-driven genes. Out of 161 MERVL–LTR-driven genes, 59 were up-regulated in Pias4-knockdown ESCs. Fold enrichment and *p*-value are shown. The *p*-value was calculated by hypergeometric test. (F) Expression of genes up-regulated in Pias4-knockdown ESCs in preimplantation mouse embryos. Center line, median; box limits, upper and lower quartiles; whiskers, 1.5× interquartile range. Preimplantation RNA-seq data are from [32]. (G) Fraction of 2C::tdTomato-positive cells in *Pias4−/−* ESCs. Left, representative fluorescence images. Right, flow cytometry analysis. Shown are mean ± SD, *n* = 3. The *p*-value was calculated by two-tailed Student’s *t* test. Scale bars, 100 μm. (H) RT-qPCR of Dux and other 2C-specific genes in Pias4-knockout ESCs. The β-actin gene was used as a control. For each gene, data were normalized to the mRNA level of wild-type ESCs. Shown are mean ± SD, *n* = 3. The *p*-value was calculated by two-tailed Student’s *t* test. (I) Western bots of PIAS4 protein level in wild-type ESCs and *Pias4−/−* ESCs rescued by empty, Pias4, or Pias4 W356A. Left, representative gel images; right, quantification of PIAS4 protein level, data were normalized to GAPDH and then to wild-type ESCs. Shown are mean ± SD, *n* = 3. (J) RT-qPCR of Dux and other 2C-specific genes in wild-type ESCs and *Pias4−/−* ESCs rescued by empty, Pias4, or Pias4 W356A. The β-actin gene was used as a control. For each gene, data were normalized to the mRNA level of wild-type ESCs. Shown are mean ± SD, *n* = 3. The *p*-value was calculated by one-way ANOVA followed by one-tailed Dunnett's test with empty versus wild-type and mutant Pias4 rescue. Source data for A, B, and G-J can be found in the supplemental data file (S1 Data). 2C, 2-cell; Dux, double homeobox; ESC, embryonic stem cell; GAPDH, glyceraldehyde 3-phosphate dehydrogenase; GSEA, gene set enrichment analysis; LTR, long terminal repeat; MERVL, murine endogenous retrovirus-L; NC, negative control; Pias4, protein inhibitor of activated STAT 4; RNA-seq, RNA sequencing; RPKM, reads per kilobase per million mapped reads; RT-qPCR, quantitative reverse transcription PCR; siRNA, small interfering RNA; SSC-H, side scatter height; Tcstv, 2C-stage variable group; tdTomato, tandem dimeric Tomato; Zfp352, zinc finger protein 352; Zscan4, zinc finger and SCAN domain containing 4.

To figure out which Sumo E3 ligase is responsible for the repression of 2C-like state and zygotic transcriptional program, we knocked down nine known Sumo E3 ligases [31] individually with small interfering RNAs (siRNAs) in 2C::tdTomato ESCs. The flow cytometry results showed that only knocking down Pias4 significantly increased the fraction of 2C-like cells (Figs 1A and S1C). Consistently, quantitative PCR (qPCR) results showed that the expression of Dux and representative 2C-specific genes is up-regulated upon knocking down Pias4 (Fig 1B). To confirm that the down-regulation of Pias4 is sufficient to globally promote the activation of zygotic transcriptional program, we performed RNA sequencing (RNA-seq) analysis on ESCs transfected with siRNAs against Pias4. The results showed that knocking down Pias4 globally increases the expression of 2C-specific ZGA transcripts (Fig 1C and S1 and S2 Tables). Importantly, a large fraction of Dux-induced genes or MERVL–long terminal repeat (LTR)-driven genes were also up-regulated in Pias4-knockdown ESCs (Fig 1D and 1E). Consistently, Pias4 knocking down also up-regulated genes induced by miR-34a knockout [13], G9a knockout [5], or LINE1 [14] or CAF-1 (p150 and p60) [8] knockdown (S1D Fig.), all of which have been shown to promote the generation of 2C-like cells. Previously, using single-cell RNA-seq, Deng and colleagues analyzed gene expression profiles during different stages of mouse preimplantation development [32]. Interestingly, we found that genes induced upon knocking down Pias4 were also up-regulated in mouse embryos during the transitioning of zygote into the 2C stage, the time when ZGA occurs (Fig 1F). These results suggest that Pias4 inhibits zygotic transcriptional program in mouse ESCs.

To exclude potential off-target effects of siRNAs, we generated Pias4-knockout ESCs (S1E Fig) using a clustered regularly interspaced short palindromic repeat (CRISPR)/CRISPR-associated protein 9 (Cas9) strategy and confirmed that knocking out Pias4 significantly increases the fraction of 2C-like cells as well as the expression of Dux and other 2C-specific genes (Figs 1G and 1H and S1F). Furthermore, we performed rescue experiment in *Pias4−/−* ESCs. We transfected *Pias4−/−* ESCs with wild-type or enzyme-dead mutant Pias4 (W356A) [33]. Western blotting analysis showed similar PIAS4 protein level between wild-type, Pias4, or Pias4 W356A rescued *Pias4−/−* ESCs (Fig 1I). qPCR results showed that wild-type but not enzyme-dead mutant Pias4 inhibited the expression of Dux and other 2C-specific genes (Fig 1J). Altogether, these data indicate that Sumo2 and Pias4 inhibit ZGA transcripts in mouse ESCs.

### PIAS4 protein is down-regulated in 2C-like cells and during ZGA

**Fig 2:**
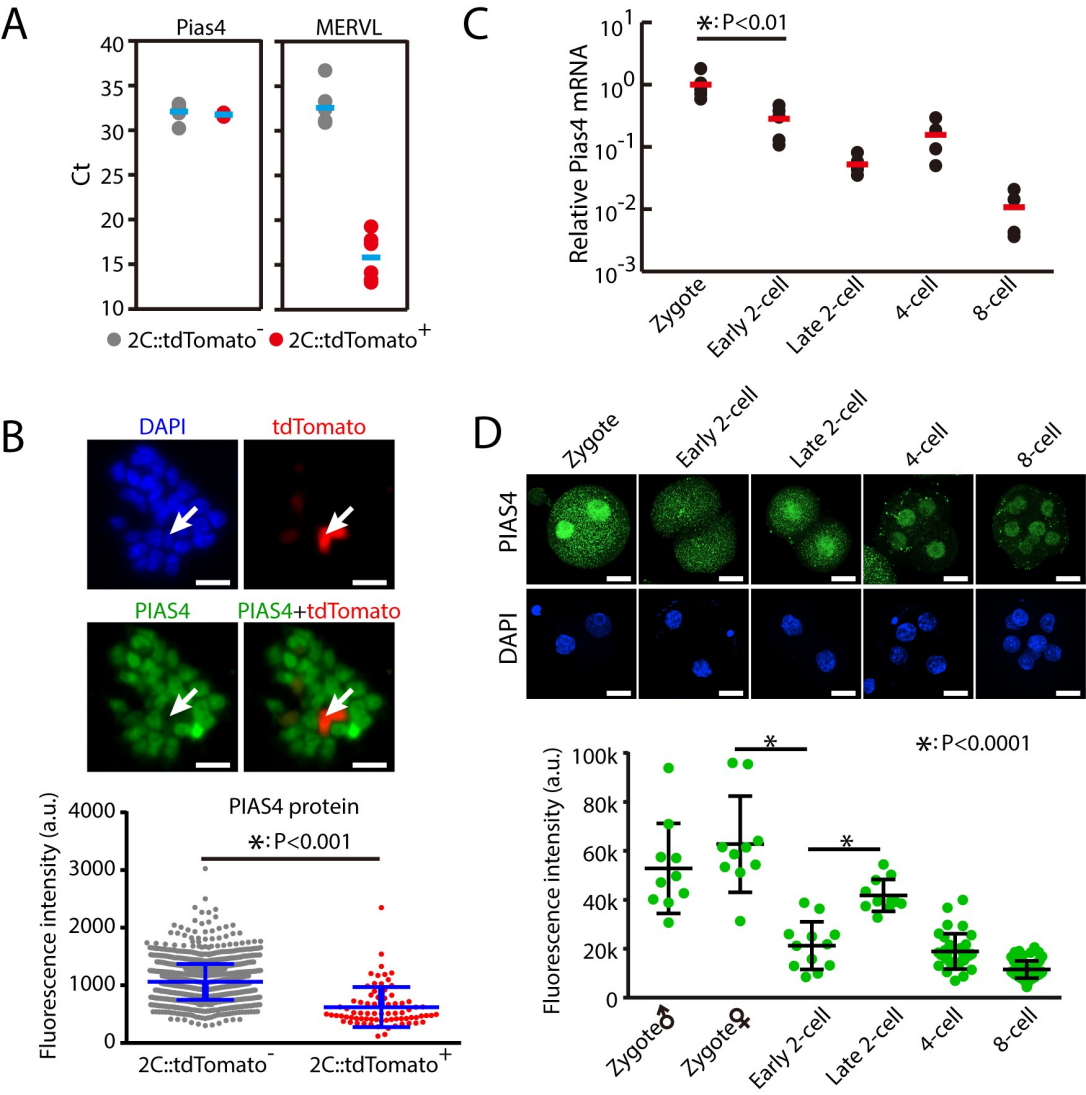
PIAS4 is down-regulated during ZGA and in 2C-like cells. (A) Single-cell RT-qPCR of Pias4 in 2C::tdTomato-positive and negative cells. Shown are Ct values. Each dot represents one cell. *n* = 6. As expected, MERVL was significantly up-regulated in 2C::tdTomato-positive cells. (B) IF staining of PIAS4 protein in 2C::tdTomato-positive and negative ESCs. Shown are representative images (left) and quantification of fluorescence intensity with mean ± SD (right). Scale bars, 20 μm. *n* = 77 tdTomato-positive cells and 1,478 tdTomato-negative cells. Each dot represents one cell. The *p*-value was calculated by two-tailed Student's *t* test. (C) Single-embryo RT-qPCR of Pias4 from zygote to the 8-cell stage. Shown is the Pias4 level normalized to zygote stage. Each dot represents one embryo. *n* = 6 for zygote, early 2C stage, and late 2C stage, and *n* = 4 for 4-cell and 8-cell stages. The *p*-value was calculated by two-tailed Student's *t* test. (D) IF staining of PIAS4 in mouse embryo from zygote to 8-cell stage. Top, representative images. Scale bars, 20 μm. Bottom, fluorescence intensity in nucleus. Shown are mean ± SD. Six to 10 embryos were analyzed at each stage. Each dot represents one nucleus. The *p*-value was calculated by one-way ANOVA followed by two-tailed Dunnett's test. Source data for A-D can be found in the supplemental data file (S1 Data). 2C, 2-cell; a.u., arbitrary units; Ct, cycle threshold; ESC, embryonic stem cell; IF, immunofluorescence; MERVL, murine endogenous retrovirus-L; Pias4, protein inhibitor of activated STAT 4; RT-qPCR, quantitative reverse transcription PCR; tdTomato, tandem dimeric Tomato; ZGA, zygotic genome activation.

Since Pias4 is an inhibitor of 2C-like state and zygotic transcriptional program, we hypothesized that the expression of Pias4 is down-regulated in 2C-like cells and during ZGA. Unexpectedly, our single-cell qPCR results showed that Pias4 mRNA level was similar between 2C::tdTomato-positive and negative cells (Fig 2A). The qPCR results are also confirmed by previous RNA-seq data (S2A Fig). Based on these results, we hypothesized that PIAS4 protein level is down-regulated in 2C-like cells. Indeed, immunofluorescence (IF) staining experiments showed that PIAS4 protein level is significantly lower in 2C::tdTomato-positive than negative cells (Fig 2B). Encouraged by these results, we further analyzed the Pias4 mRNA and protein level during early embryo development. Results from single-embryo quantitative reverse transcription PCR (RT-qPCR) showed that Pias4 mRNA was significantly decreased from zygote to the 2C embryo stage (Fig 2C), coincident with the up-regulation of ZGA transcripts including Dux, MERVL, and Zscan4d (S2B Fig). Consistent with qPCR results, IF staining showed that Pias4 protein was also significantly down-regulated during ZGA (Fig 2D). These results show that PIAS4 protein is down-regulated in 2C-like cells and during ZGA, consistent with its inhibitory role in zygotic transcriptional program. However, whereas both RNA and protein level of Pias4 are down-regulated during ZGA, only protein level of Pias4 is down-regulated in 2C-like cells, indicating different control mechanisms for Pias4 between early development in vivo and ESCs cultured in vitro.

### Overexpression of Pias4 inhibits the zygotic transcriptional program and impairs early embryo development

**Fig 3:**
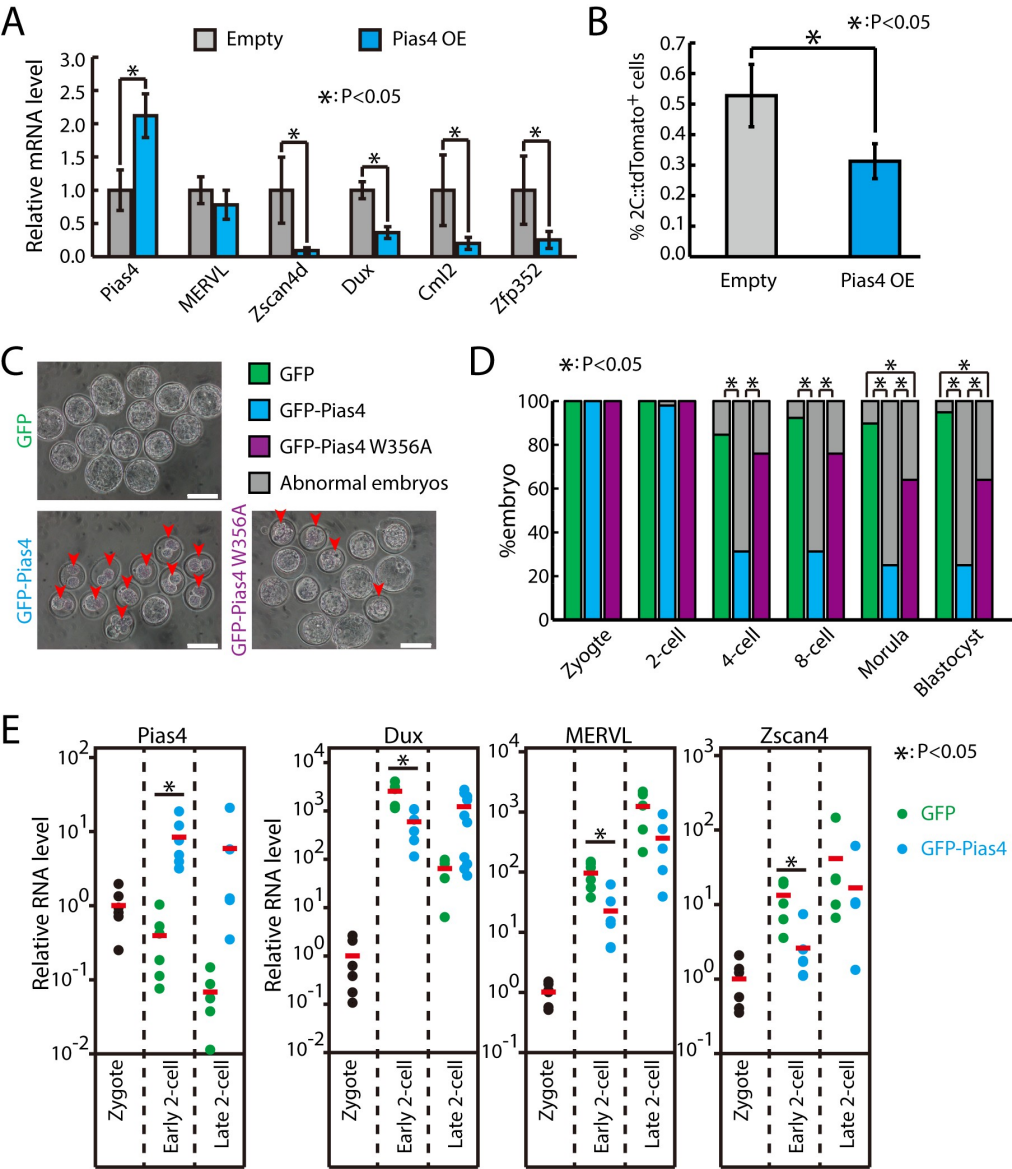
Pias4 OE inhibits zygotic transcriptional program and impairs early embryo development. (A) RT-qPCR of Dux and other 2 cell–specific genes in control and Pias4-overexpressing ESCs. The β-actin gene was used as a control. For each gene, data were normalized to the mRNA level of control OE ESCs. Shown are mean ± SD, *n* = 3. The *p*-value was calculated by two-tailed Student’s *t* test. (B) Fraction of 2C::tdTomato-positive cells in control and Pias4-overexpressing ESCs. Data are presented as mean ± SD, *n* = 3. The *p*-value was calculated by two-tailed Student’s *t* test. (C) Development of zygotes that were injected with in vitro transcribed GFP, GFP–Pias4, and GFP–Pias4 W356A mRNAs. Shown are representative images at development time equivalent to blastocyst stage. Arrows point to abnormal embryos. Scale bars, 100 μm. (D) Development of zygote injected with GFP, GFP–Pias4, and GFP–Pias4 W356A mRNAs. Shown is the percentage of normally developed embryos. *n* = 39, 48, and 25 for GFP, GFP–Pias4, and GFP–Pias4 W356A mRNA–transfected embryos from three independent experiments, respectively. The *p*-value was calculated by Pearson's chi-squared test. (E) Single-embryo RT-qPCR of embryos injected with GFP or GFP–Pias4 mRNA in early and late 2-cell stages. Data were normalized to zygote stage. Each dot represents one embryo. Red bars indicate mean value. *n* = 6 for zygote, early 2-cell and *n* = 5 for late 2-cell. The *p*-value was calculated by two-tailed Student’s *t* test. Source data for A, B, D, and E can be found in the supplemental data file (S1 Data). Dux, double homeobox; ESC, embryonic stem cell; GFP, green fluorescent protein; MERVL, murine endogenous retrovirus-L; OE, overexpression; Pias4, protein inhibitor of activated STAT 4; RT-qPCR, quantitative reverse transcription PCR; tdTomato, tandem dimeric Tomato; Zfp352, zinc finger protein 352; Zscan4d, zinc finger and SCAN domain containing 4D.

Next, we tested whether Pias4 is sufficient to repress the zygotic transcriptional program by overexpressing Pias4 in ESCs (Fig 3A). qPCR results showed that around 2-fold overexpression (OE) of Pias4 could effectively decrease the expression of Dux and other 2C-specific genes (Fig 3A). Consistent with the repression of zygotic transcriptional program, the percentage of 2C-like cells was also significantly decreased upon Pias4 OE (Fig 3B). To test whether Pias4 plays a similar inhibitory role during ZGA, we ectopically expressed Pias4 in mouse zygotes. In agreement with findings in ESCs, injecting in vitro transcribed green fluorescent protein (GFP)–Pias4 but not GFP or GFP–Pias4 W356A into zygotes induced embryonic arrest at the 2C stage (Fig 3C and 3D). Importantly, single-embryo RT-qPCR results showed that the up-regulation of Dux, MERVL, and Zscan4d were significantly inhibited in embryos injected with GFP–Pias4 at the early phase of the 2C stage (Fig 3E). Moreover, Dux expression was only transiently activated at early 2C stage in GFP-injected embryos and quickly down-regulated at the late 2C stage (Fig 3E). In contrast, the rapid down-regulation of Dux was not observed in Pias4-injected embryos (Fig 3E), suggesting that ZGA progression is severely interfered with by Pias4 OE. Together, these results demonstrate the potent function of Pias4 in inhibiting the transition of ESCs into 2C-like cells and ZGA processes during early embryonic development.

### Knocking down Pias4 decreases the sumoylated protein form of multiple activators and inhibitors of zygotic transcriptional program

To figure out how PIAS4 inhibits zygotic transcriptional program, we decided to identify downstream substrate proteins of PIAS4. To this end, we performed pull-down of Sumo2 modified proteins followed by mass spectrometry (MS) analysis in control or Pias4 siRNAs–transfected ESCs, based on an assumption that the abundance of Pias4 substrates in Sumo2 pull-down should decrease upon Pias4 knockdown. We identified 97 potential candidate substrate proteins whose sumoylated form was decreased ≥1.25-fold in Pias4-knockout ESCs (S3A Fig and S3 Table). The decrease of sumoylated protein form might be caused by a decrease in sumoylation efficiency due to Pias4 knockout; alternatively, this could be due to the decrease of protein expression upon Pias4 knockout. These targets are significantly enriched for Gene Ontology (GO) terms like transcription or regulation of transcription (S3B Fig), supporting the function of sumoylation mediated by Pias4 in regulating transcriptional program. To identify the downstream target(s) of PIAS4 in regulating zygotic transcriptional program, we performed a CRISPR interference (CRISPRi) [34] screening in Zscan4::GFP reporter ESCs [4,7] (S3C Fig). Knocking down an inhibitor of 2C transcriptional program should lead to the increase of Zscan4::GFP-positive cells, whereas knocking down an activator of 2C transcriptional program should lead to the decrease of Zscan4::GFP-positive cells. Moreover, knocking down an activator downstream of Pias4 should diminish the effect of Pias4 siRNAs. Therefore, we also performed CRISPRi screening in the presence of Pias4 siRNAs (S3C Fig), hoping to identify direct activators of 2C transcriptional program downstream of Pias4. The results from these experiments suggest that mitochondrial ribosomal protein L38 (Mrpl38), L-threonine dehydrogenase (Tdh), and Zfp131 are potential inhibitors, and Dppa2, Nop58, protein arginine N-methyltransferase 5 (Prmt5), and nucleus accumbens associated 1 (Nacc1) are potential activators of zygotic transcriptional program in ESCs (S3C Fig). As we were more interested in the activators of Dux and zygotic transcriptional program, we further verified the function of potential activators including Dppa2, Nop58, Prmt5, and Nacc1 in 2C::tdTomato reporter cells with siRNAs against selected targets. The results confirmed that Dppa2, Nop58, Prmt5, and Nacc1 are activators of zygotic transcriptional program in ESCs (S3D Fig). Among them, knocking down Dppa2 showed the strongest inhibition effect in the emergence of 2C-like cells (S3D Fig). Together, these data suggest that PIAS4 might regulate the sumoylation (Sumo2) of multiple inhibitors and activators of zygotic transcriptional program.

### Dppa2 and Dppa4 are essential for activating zygotic transcriptional program in mouse ESCs

**Fig 4:**
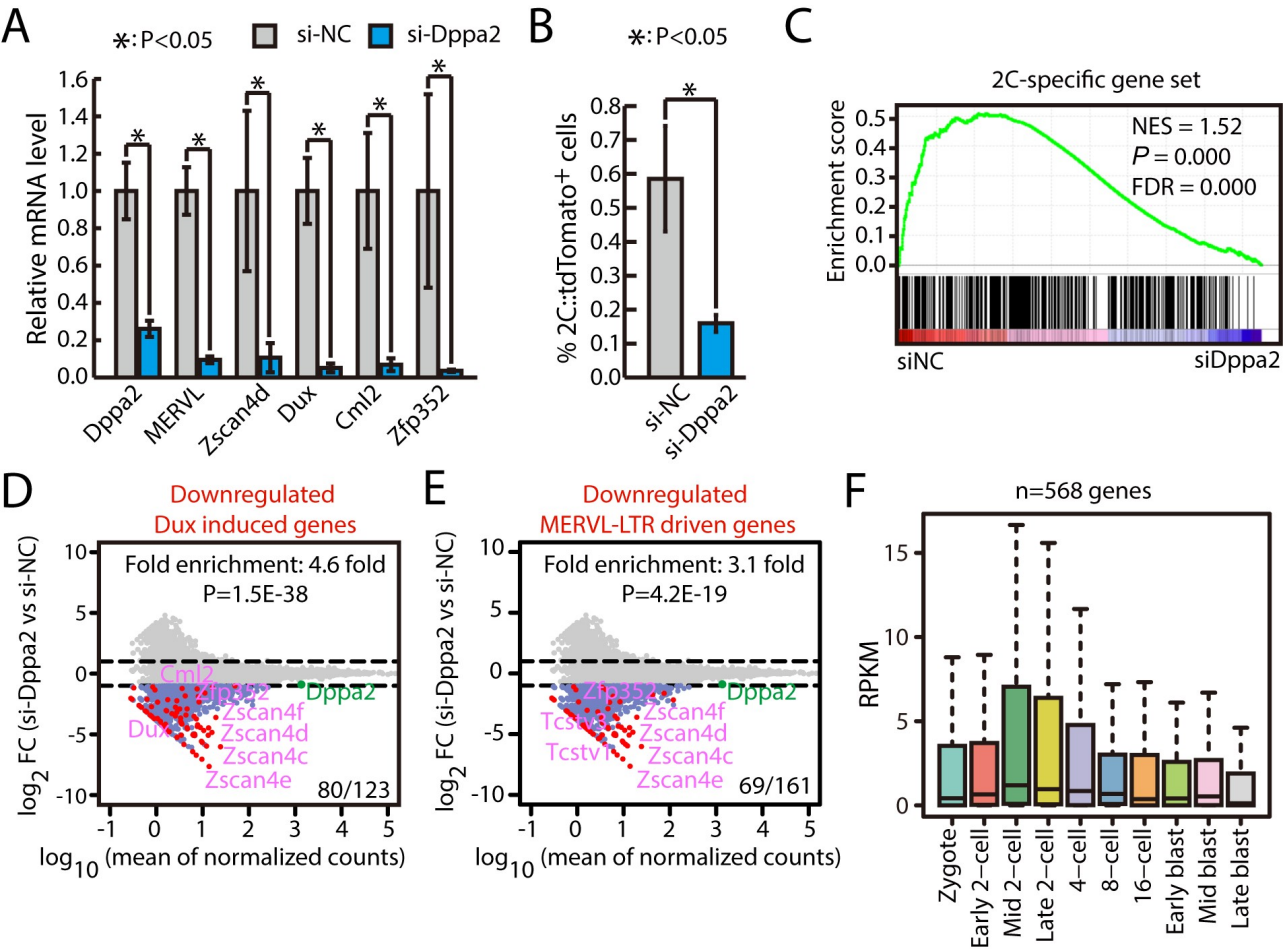
Dppa2 is essential for the activation of zygotic transcriptional program in mouse ESCs. (A) RT-qPCR of Dppa2, Dux, and other 2C-like ZGA genes in ESCs treated with siRNA against Dppa2. The β-actin gene was used as a control. For each gene, data were normalized to the mRNA level of wild-type ESCs. Shown are mean ± SD, *n* = 3. The *p*-value was calculated by two-tailed Student’s *t* test. (B) Fraction of 2C::tdTomato-positive cells in ESCs treated with siRNA against Dppa2. Data are presented as mean ± SD, *n* = 3. The *p*-value was calculated by two-tailed Student’s *t* test. (C) GSEA for 2C-specific genes in ESCs transfected with control siRNAs or siRNAs against Dppa2. For x-axis, genes were ranked based on the ratio of siNC versus si-Dppa2 ESCs. (D) MA plots showing gene expression changes in Dppa2-knockdown ESCs. Red dots indicate Dux-induced genes. Out of 123 Dux-induced genes, 80 were down-regulated in Dppa2-knockdown ESCs. Fold enrichment and *p*-value are shown. The *p*-value was calculated by hypergeometric test. (E) MA plots showing changes in genes in Dppa2-knockdown ESCs. Red spots were MERVL–LTR-driven genes. Out of 161 MERVL–LTR-driven genes, 69 were down-regulated in Dppa2-knockdown ESCs. Fold enrichment and *p*-value are shown. The *p*-value was calculated by hypergeometric test. (F) Expression of genes down-regulated in Dppa2-knockdown ESCs in preimplantation mouse embryos. Center line, median; box limits, upper and lower quartiles; whiskers, 1.5× interquartile range. Preimplantation RNA-seq data are from [32]. Source data for A and B can be found in the supplemental data file (S1 Data). 2C, 2-cell; Dppa2, developmental pluripotency associated 2; Dux, double homeobox; ESC, embryonic stem cell; FC, fold change; FDR, false discovery rate; GSEA, gene set enrichment analysis; LTR, long terminal repeat; MERVL, murine endogenous retrovirus-L; NES, normalized enrichment score; RNA-seq, RNA sequencing; RT-qPCR, quantitative reverse transcription PCR; siNC, siRNA against negative control; siRNA, small interfering RNA; tdTomato, tandem dimeric Tomato; Zfp352, zinc finger protein 352; ZGA, zygotic genome activation; Zscan4, zinc finger and SCAN domain containing 4.

Next, we focused our study on Dppa2, a transcription factor expressed in germinal vesicle (GV) oocytes [35], whose dominant negative form arrests early mouse embryo development [36]. We transfected 2C::tdTomato reporter ESCs with siRNAs against Dppa2. The qPCR results showed that knocking down Dppa2 significantly decreased the expression of Dux and other 2C-specific genes (Fig 4A), consistent with the decrease in the percentage of 2C-like ESCs (Fig 4B). To confirm that Dppa2 is responsible for the up-regulation of zygotic transcriptional program upon Pias4 knockdown, we cotransfected siRNAs against Dppa2 and Pias4. The results show that knocking down Dppa2 can effectively prevent the up-regulation of Dux and other 2C-like ZGA genes upon Pias4 knockdown (S4A Fig). Since Dppa4 has been shown to play similar roles as Dppa2 in the activation of Dux and ZGA program [15, 16], we also tested the effect of knocking down Dppa4 in ESCs. Indeed, qPCR results showed that knocking down Dppa4 decreased the expression of Dux and other 2C-specific genes at an extent similar to Dppa2 knockdown (S4B Fig). In addition, double knockdown of Dppa2 and Dppa4 caused a similar degree of down-regulation for Dux and Cml2 and further down-regulation of Zscan4d and Zfp352 (S4B Fig). We further performed RNA-seq for ESCs with Dppa2 and Dppa4 knocked down individually or in combination. The results confirmed that both Dppa2 and Dppa4 are essential for the expression of ZGA transcripts (Figs 4C–4F and S5A–S5D and S4 Table). We found 50 2C-specific genes down-regulated ≥50% in double knockdown versus control. Among them, 31 genes were similarly down-regulated (differences within 20% range) in siDppa2, siDppa4, or siDppa2+siDppa4. Interestingly, six genes were further down-regulated ≥20% in double knockdown than any of the single knockdown. These results suggest that the function of Dppa2 and Dppa4 is largely cooperative in regulating the expression of 2C-specific genes, but another mode of action (e.g., redundant) likely exists. In addition, the overall profiles for knocking down Dppa2, Dppa4, or both were largely similar (S6A Fig). Intriguingly, Dppa2 and Dppa4 knockdown also down-regulated genes induced by other conditions that promoted a 2C-like state, including miR-34a knockout, G9a knockout, and LINE1 or CAF-1 knockdown (S6B Fig). Together, these experiments support that Dppa2 and Dppa4 are essential for activating zygotic transcriptional program in mouse ESCs.

### The sumoylation of DPPA2 by PIAS4 depends on K31 and K108 sites

**Fig 5:**
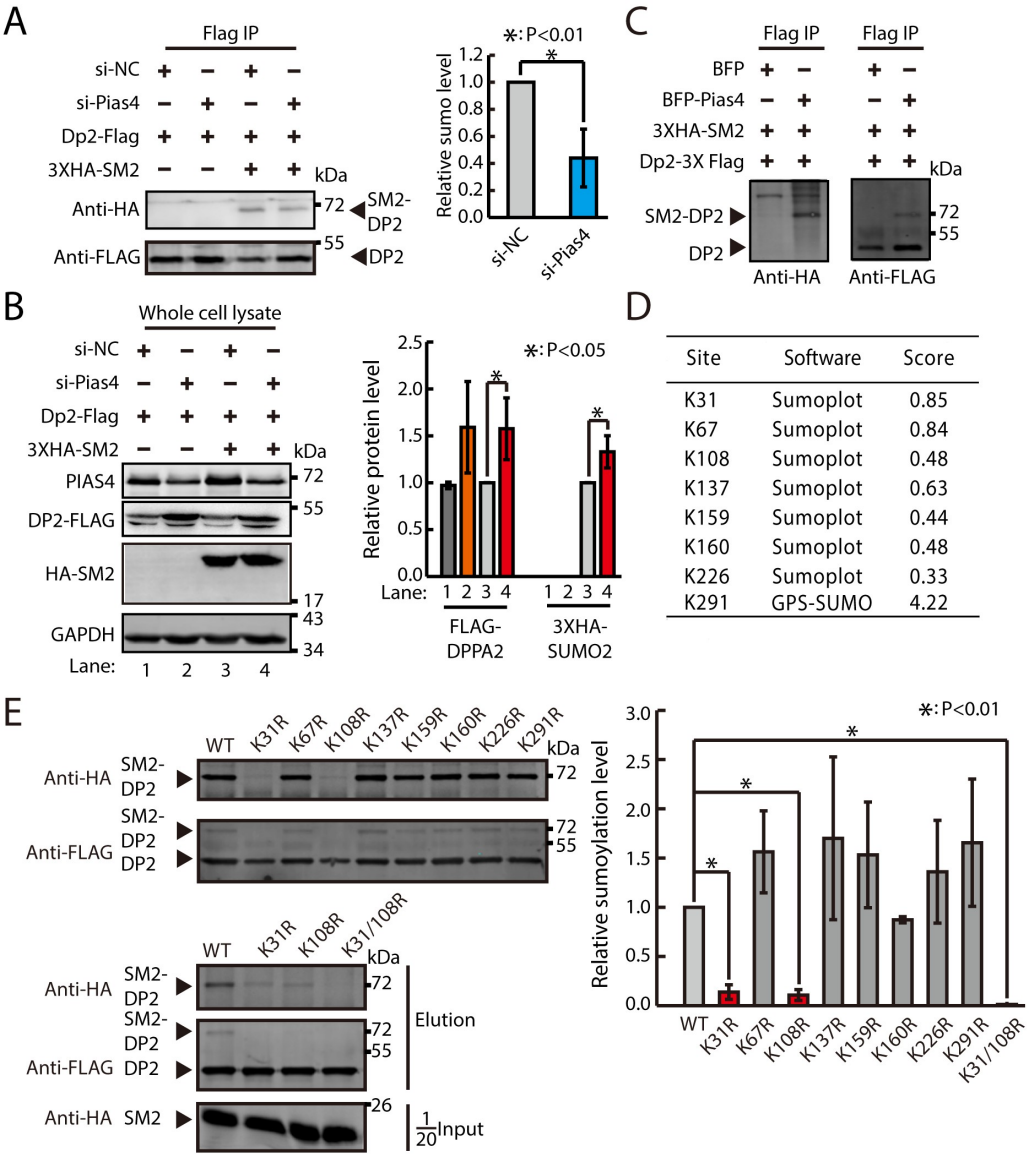
The sumoylation of DPPA2 by PIAS4 depends on K31 and K108 sites. (A) Western blotting analysis of Flag IP samples in ESCs overexpressing Dppa2–3XFlag and 3XHA–Sumo2 and treated with control or Pias4 siRNAs. Quantification of relative sumoylation level is shown below. Data were normalized to siNC-treated ESCs overexpressing Dppa2–3XFlag and 3XHA–Sumo2. Shown are mean ± SD, *n* = 3. The *p*-value was calculated by two-tailed Student’s *t* test. (B) Western blotting analysis of FLAG–DPPA2 and 3XHA–SUMO2 in ESCs transfected with siNCs or Pias4. Left, representative images of gel blots; right, quantification of FLAG–DPPA2 and 3XHA–SUMO2. Data were normalized to GAPDH and then to Flag–Dppa2/3XHA–Sumo2 overexpressing ESCs treated with siNC. Shown are mean ± SD, *n* = 3. The *p*-value was calculated by two-tailed Student’s *t* test. (C) Western blotting analysis of Flag IP samples in HEK293T cells transfected with Dppa2–3XFlag and 3XHA–Sumo2 with or without overexpression of Pias4. (D) Table of the predicted SUMOylation sites using in silico prediction programs SUMOplot and GPS-SUMO in DPPA2. Scores ware based on two criteria: direct amino acid match to SUMO consensus motif (ψKXE) or substitution of the consensus amino acid residues with amino acid residues exhibiting similar hydrophobicity. (E) Western blotting analysis of Flag IP samples in HEK293T cells transfected with flag-tagged lysine mutant, 3XHA-Sumo2, and Pias4. Quantification for relative sumoylation level is shown below. Data were normalized to HEK293T cells transfected with flag-tagged WT Dppa2, 3XHA-Sumo2, and Pias4. Shown are mean ± SD, *n* = 3. The *p*-value was calculated by one-way ANOVA followed by two-tailed Dunnett's test. Source data for A, B, and E can be found in the supplemental data file (S1 Data). BFP, blue fluorescent protein; Dppa2, developmental pluripotency associated 2; ESC, embryonic stem cell; GAPDH, glyceraldehyde 3-phosphate dehydrogenase; HA, hemagglutinin; HEK293T, human embryonic kidney 293T; IP, immunoprecipitation; PIAS4, protein inhibitor of activated STAT 4; siNC, siRNA against negative control; siRNA, small interfering RNA; SUMO, small ubiquitin-like modifier; WT, wild type.

Next, we verified that PIAS4 can catalyze the sumoylation of DPPA2 in mouse ESCs (Fig 5A). We overexpressed Flag–Dppa2 and hemagglutinin (HA)–Sumo2 in ESCs treated with siRNAs against negative control (siNCs) or Pias4. As expected, knocking down Pias4 decreased the fraction of sumoylated DPPA2 (Fig 5A). In addition, FLAG–DPPA2 and HA–SUMO2 were slightly but significantly increased in Pias4-knockdown ESCs (Fig 5B), suggesting that the decrease in the fraction of sumoylated DPPA2 is due to the decrease in sumoylation activity but not the expression of DPPA2 or HA–SUMO2 proteins in Pias4-knockdown ESCs. Furthermore, overexpressing PIAS4 increased the fraction of sumoylated DPPA2 in human embryonic kidney 293T (HEK293T) cells (Fig 5C). To identify which lysine residues are important for the sumoylation of DPPA2 by PIAS4, we decided to map the potential sumoylation sites on DPPA2 protein. Using the in silico prediction programs SUMOplot (Abgent) and GPS-SUMO (The Cuckoo Workgroup), eight lysine residues (K31, K67, K108, K137, K159, K160, K226, and K291) were predicted as possible candidate sites for SUMO-conjugation in DPPA2 (Fig 5D). Mutant proteins for all predicted lysine residues were then generated, and their effects on sumoylation of DPPA2 were examined in HEK293T cells. K31R and K108R mutations led to a substantial reduction in the intensity of the higher-molecular-mass sumoylated DPPA2 bands (Fig 5E). These results suggest that the sumoylation of DPPA2 by PIAS4 depends on K31 and K108 sites.

### Sumo2–Dppa2 inhibits the expression of 2C-specific genes and impairs early embryo development

**Fig 6:**
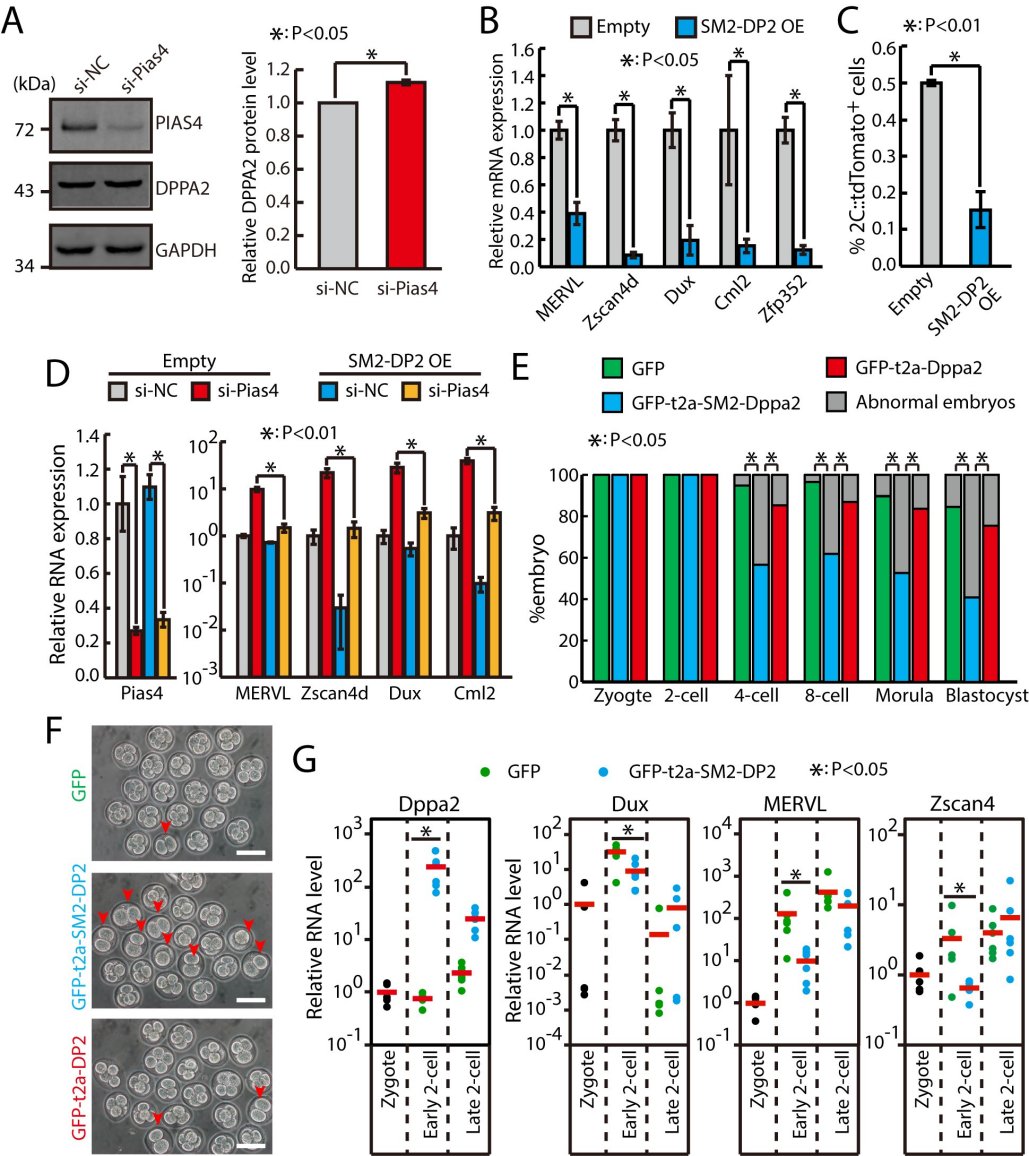
Sumo2–Dppa2 (“SM2-DP2”) inhibits zygotic transcriptional program and impairs early embryo development. (A) Western blotting analysis of DPPA2 in ESCs treated with control and Pias4 siRNAs. Shown are mean ± SD, *n* = 3. (B) RT-qPCR of Dux and other 2C-specific genes in control and Sumo2ΔGG–Dppa2 overexpressing ESCs. The β-actin gene was used as a control. For each gene, data were normalized to the mRNA level of control OE ESCs. Shown are mean ± SD, *n* = 3. The *p*-value was calculated by two-tailed Student’s *t* test. (C) Fraction of 2C::tdTomato-positive cells in control and Sumo2ΔGG–Dppa2-overexpressing ESCs. Data are presented as mean ± SD, *n* = 3. The *p*-value was calculated by two-tailed Student’s *t* test. (D) RT-qPCR analysis of Dux and ZGA transcripts in control and Sumo2ΔGG–Dppa2-overexpressing ESCs treated with control and Pias4 siRNAs. Data are normalized to β-actin and wild-type siNC. Shown are presented as mean ± SD, *n* = 3. The *p*-value was calculated by two-way ANOVA followed by two-tailed Dunnett's test. (E) Development of zygote injected with GFP, GFP–t2a–Dppa2, and GFP–t2a–Sumo2ΔGG–Dppa2 mRNAs. Shown is the percentage of normally developed embryos. *n* = 58, 76, and 61 embryos for GFP, GFP–t2a–SM2–Dppa2, and GFP–t2a–Dppa2 from four experiments, respectively. The *p*-value was calculated by Pearson's chi-squared test. (F) Development of zygotes that were injected with in vitro transcribed GFP, GFP–t2a–Dppa2, and GFP–t2a–Sumo2ΔGG–Dppa2 mRNAs. Shown are representative images for development time equivalent to the 4-cell stage. Arrows point to abnormal embryos remained at the 2C stage. Scale bars, 100 μm. (G) Single-embryo RT-qPCR of Dppa2, Dux, MERVL, and Zscan4 in embryos injected with Sumo2ΔGG–Dppa2 mRNA. Data were normalized to zygote stage. Each dot represents one embryo. Red bars indicate mean value. *n* = 5 for zygote and *n* = 6 for early and late 2C-stage embryos. The *p*-value was calculated by one-tailed Student’s *t* test. Source data for A-E and G can be found in the supplemental data file (S1 Data). 2C, 2-cell; Dppa2, developmental pluripotency associated 2; Dux, double homeobox; ESC, embryonic stem cell; GAPDH, glyceraldehyde 3-phosphate dehydrogenase; GFP, green fluorescent protein; MERVL, murine endogenous retrovirus-L; OE, overexpression; Pias4, protein inhibitor of activated STAT 4; RT-qPCR, quantitative reverse transcription PCR; siNC, siRNA against negative control; siRNA, small interfering RNA; Sumo2, small ubiquitin-like modifier 2; tdTomato, tandem dimeric Tomato; Zfp352, zinc finger protein 352; ZGA, zygotic genome activation; Zscan4d, zinc finger and SCAN domain containing 4D.

To figure out the impact of sumoylation by Pias4 on the protein level of DPPA2, we performed western analysis of DPPA2 protein in control siRNA or ESCs treated with Pias4 siRNA. The results showed that the DPPA2 protein was only slightly increased upon Pias4 knockdown (Fig 6A), indicating that Pias4 may regulate zygotic transcriptional program through regulating the sumoylation but not the protein level of DPPA2. In order to test the consequence of sumoylation of DPPA2 by PIAS4, a DPPA2 fused with SUMO2ΔGG at the N terminal was ectopically expressed in ESCs to mimic the sumoylated DPPA2 [37,38]. To avoid artifacts due to high expression level, SUMO2ΔGG–DPPA2 was expressed at a significantly lower level than endogenous DPPA2 protein (S7A and S7B Fig). Interestingly, we found that expressing SUMO2ΔGG–DPPA2 at this level can significantly decrease the expression of 2C-specific genes as well as the fraction of 2C-like cells (Fig 6B and 6C). Moreover, SUMO2ΔGG-DPPA2 almost completely abolished the increase of the fraction of 2C-like cells and the up-regulation of Dux and other 2C-specific genes in Pias4-knockdown ESCs (Figs 6D and S7C), supporting the statement that sumoylated DPPA2 functions as an inhibitor of zygotic transcriptional program downstream of Pias4. Finally, overexpressing SUMO2ΔGG–DPPA2 at a much lower level still caused similar repression of Dux and other 2C-specific genes and similar decrease in the fraction of 2C-like cells (S7D–S7F Fig). Since sumoylated DPPA2 has an inhibitory role for the zygotic transcriptional program, we reasoned that sumoylated DPPA2 must decrease in 2C::tdTomato-positive cells. To test this, we performed a proximity ligation assay (PLA) [39] using antibodies against SUMO2 and DPPA2. Indeed, the PLA assay results showed that the signal of colocalization of SUMO2 and DPPA2 is lower in 2C::tdTomato-positive cells (S7G Fig). Meanwhile, the protein level of DPPA2 is significantly increased in 2C::tdTomato-positive cells with no apparent alteration of localization (S7H and S7I Fig). Finally, we tested the inhibitory role of sumoylated DPPA2 for zygotic transcriptional program in mouse embryos. Compared to GFP and GFP–T2A–DPPA2, the expression of GFP–T2A–SUMO2ΔGG–DPPA2 in zygote significantly impaired the early embryo development (Fig 6E and 6F), accompanied with insufficient up-regulation of 2C-specific genes at the early phase of the 2C stage (Fig 6G). Therefore, sumoylated DPPA2 likely plays a dominant negative function to DPPA2. Taken together, these data showed that PIAS4 inhibits zygotic transcriptional program at least partially through sumoylation of DPPA2.

### OE of Dppa2 activates zygotic transcriptional program

**Fig 7:**
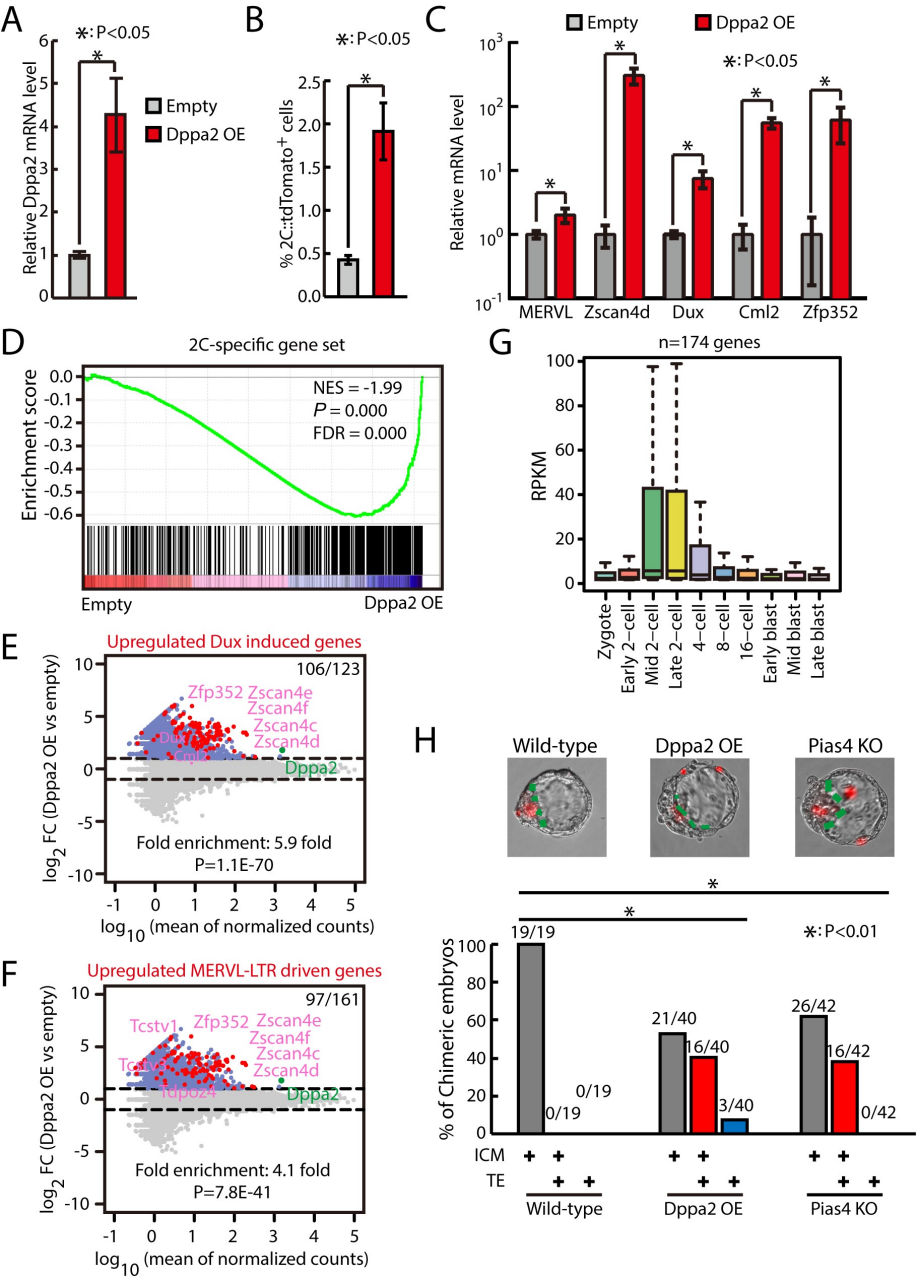
Dppa2 OE activates zygotic transcriptional program. (A) RT-qPCR of Dppa2 in control and Dppa2-overexpressing ESCs. The β-actin gene was used as a control. Data were normalized to the mRNA level of control ESCs. Shown are mean ± SD, *n* = 3. The *p*-value was calculated by two-tailed Student’s *t* test. (B) Fraction of 2C::tdTomato-positive cells in control and Dppa2-overexpressing ESCs. Data are presented as mean ± SD, *n* = 3. The *p*-value was calculated by two-tailed Student’s *t* test. (C) RT-qPCR of Dux and other 2C-specific genes in control and Dppa2-overexpressing ESCs. The β-actin gene was used as a control. For each gene, data were normalized to the mRNA level of control ESCs. Shown are mean ± SD, *n* = 3. The *p*-value was calculated by two-tailed Student’s *t* test. (D) GSEA for 2C-specific genes in control and Dppa2-overexpressing ESCs. For x-axis, genes were ranked based on the ratio of control versus Dppa2-overexpressing ESCs. (E) MA plots showing gene expression changes in Dppa2-overexpressing ESCs. Red dots indicate Dux-induced genes. Out of 123 Dux-induced genes, 106 were up-regulated in Dppa2-overexpressing ESCs. Fold enrichment and *p*-value are shown. The *p*-value was calculated by hypergeometric test. (F) MA plots showing gene expression changes in Dppa2-overexpressing ESCs. Red dots indicate MERVL–LTR-driven genes. Out of 161 MERVL–LTR-driven genes, 97 were up-regulated in Dppa2-overexpressing ESCs. Fold enrichment and *p*-value are shown. The *p*-value was calculated by hypergeometric test. (G) Expression of up-regulated genes in Dppa2-overexpressing ESCs in preimplantation mouse embryos. Center line, median; box limits, upper and lower quartiles; whiskers, 1.5× interquartile range. Preimplantation RNA-seq data are from [32]. (H) Wild-type, Dppa2-overexpressing, and *Pias4−/−* ESCs expressing mRuby2 fluorescence protein were cocultured with 8-cell embryo, and their contribution to ICM and TE were determined by the localization of mRuby2-positive mouse ESCs. Top, representative images. Bottom, the percentage of chimeric blastocyst embryos with ESC contribution to ICM, TE, and ICM+TE. Number of embryos is indicated. The *p*-value was determined by Fisher's exact test. Source data for A-C and H can be found in the supplemental data file (S1 Data). 2C, 2-cell; Dppa2, developmental pluripotency associated 2; Dux, double homeobox; ESC, embryonic stem cell; FC, fold change; FDR, false discovery rate; GSEA, gene set enrichment analysis; ICM, inner cell mass; KO, knockout; LTR, long terminal repeat; MERVL, murine endogenous retrovirus-L; NES, normalized enrichment score; OE, overexpression; Pias4, protein inhibitor of activated STAT 4; RNA-seq, RNA sequencing; RPKM, reads per kilobase per million mapped reads; RT-qPCR, quantitative reverse transcription PCR; siRNA, small interfering RNA; tdTomato, tandem dimeric Tomato; TE, trophectoderm; Zfp352, zinc finger protein 352; ZGA, zygotic genome activation; Zscand4, zinc finger and SCAN domain containing 4.

We then tested whether OE of Dppa2 is sufficient to activate zygotic transcriptional program. The flow cytometry analysis showed that around 4-fold OE of Dppa2 (Fig 7A) significantly increased the fraction of 2C::tdTomato-positive cells (Fig 7B). In addition, Dppa2 OE further increased the fraction of 2C-like cells in Sumo2- or Pias4-knockdown ESCs (S8A Fig). Importantly, qPCR results showed that Dppa2 significantly activated the expression of Dux (around 7-fold) and other 2C-specific genes (Fig 7C). To find out the global impact of Dppa2 OE on zygotic transcriptional program, we performed RNA-seq in Dppa2-overexpressing ESCs. The results showed that Dppa2 OE globally up-regulated the expression of 2C-specific ZGA transcripts as well as Dux-induced genes and MERVL–LTR-driven genes (Fig 7D–7F and S5 and S6 Table). In addition, Dppa2 OE also up-regulated genes induced by CAF-1 knockdown, LINE1 knockdown, G9a knockout, and mir-34a knockout (S8B Fig). Moreover, genes induced by Dppa2 OE were also up-regulated in mouse embryos during the transitioning of zygote [32] into the 2C stage, when ZGA takes place (Fig 7G). Interestingly, PCA analysis showed that Dppa2-OE or Pias4-knockdown ESCs clustered closely with other 2C-like cells including P150-, P60-, or LINE1-knockdown ESCs (S8C Fig). To gather more evidence that Dppa2 OE and *Pias4−*/*−* ESCs have properties of 2C-like cells, we performed chimera formation by aggregating ESCs with 8-cell embryos. The results showed that Dppa2 OE and *Pias4−*/*−* ESCs produced chimeric blastocysts with incorporation into both inner cell mass (ICM) and trophectoderm (TE) at a significantly higher frequency than wild-type ESCs (Fig 7H). Together, these results indicate that overexpressing Dppa2 is sufficient to activate the expression of ZGA transcripts.

Next, we tested whether Dppa4 OE can activate the zygotic transcriptional program. Interestingly, qPCR results showed that Dppa4 alone cannot activate the expression of Dux and other 2C-specific genes. In addition, OE of Dppa4 slightly increased the expression of Dux and other 2C-specific genes in Dppa2-overexpressing ESCs (S8D Fig). However, the fraction of 2C-like cells was significantly increased in Dppa2/4-overexpressing versus Dppa2-overexpressing ESCs (S8E Fig). Together, these results support that OE of Dppa2 activates the zygotic transcriptional program and Dppa4 can enhance the function of Dppa2.

### Dppa2 activates zygotic transcriptional program through directly activating Dux

**Fig 8:**
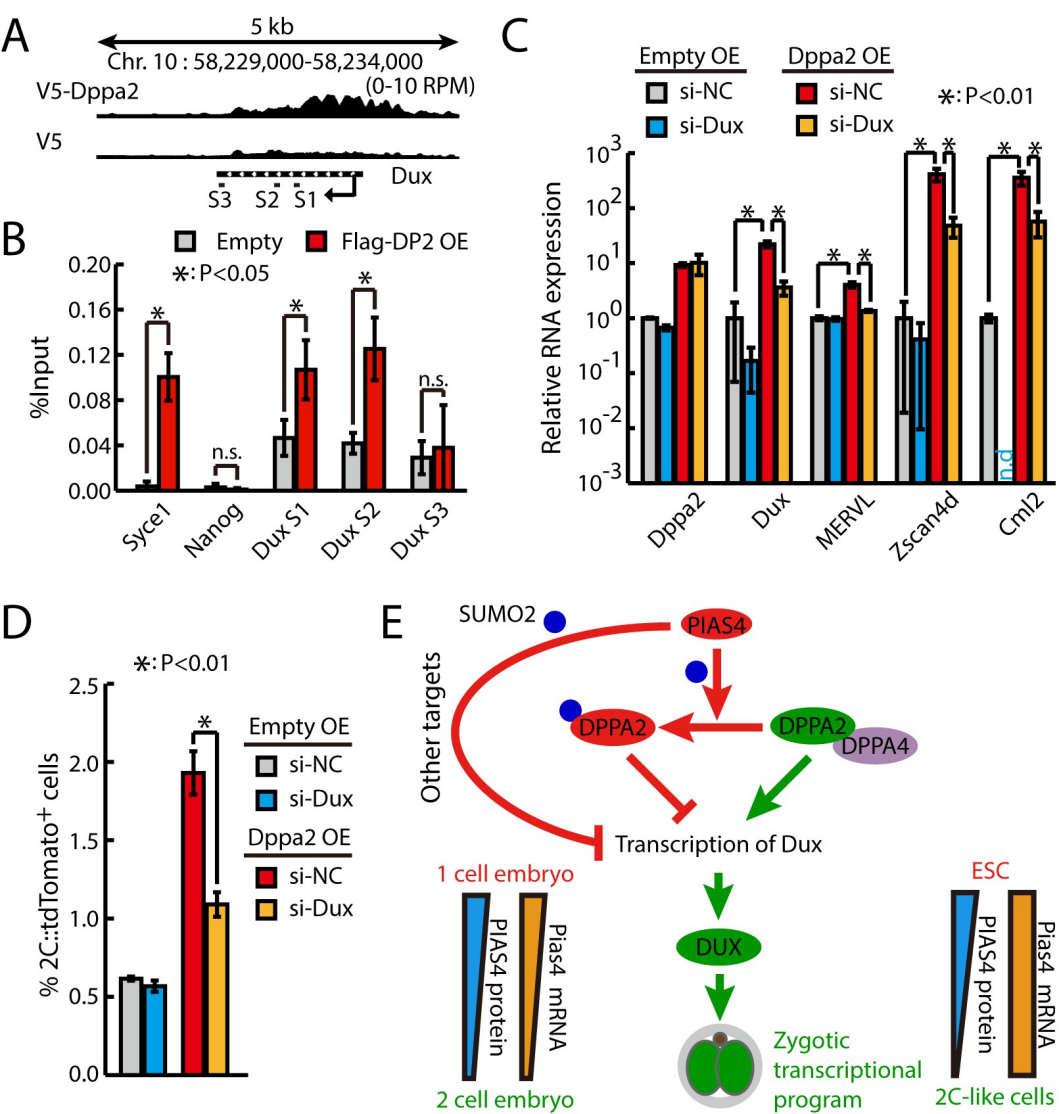
Dppa2 (“DP2”) activates 2C-like program through directly activating Dux. (A) Screenshot of V5–Dppa2 ChIP-seq tracks at Dux locus. Primer sets for ChIP-qPCR are indicated. (B) Flag ChIP-qPCR of control and Dppa2–3xFlag-overexpressing ESCs at *Dux* locus. Data were normalized to input. Data are presented as mean ± SD, *n* = 3. The *p*-value was calculated by two-tailed Student’s *t* test. *Syce1* and *Nanog* serving as positive and negative controls, respectively. (C) RT-qPCR of Dux and other 2C-specific genes in control and Dppa2-overexpressing ESCs treated with siRNAs against Dux. The β-actin gene was used as a control. For each gene, data were normalized to the mRNA level of control ESCs treated with siNCs. Shown are mean ± SD, *n* = 3. The *p*-value was calculated by two-way ANOVA followed by two-tailed Dunnett's test. (D) Fraction of 2C::tdTomato-positive cells in control and Dppa2-overexpressing ESCs treated with siRNAs against Dux. Data are presented as mean ± SD, *n* = 3. The *p*-value was calculated by two-way ANOVA followed by two-tailed Dunnett's test. (E) Summary graph showing the opposing function of Dppa2/4 and Pias4 in the regulation of zygotic transcriptional program. Source data for B-D can be found in the supplemental data file (S1 Data). 2C, 2-cell; ChIP-seq, chromatin immunoprecipitation sequencing; Dppa2/4, developmental pluripotency associated 2 and 4; Dux, double homeobox; ESC, embryonic stem cell; MERVL, murine endogenous retrovirus-L; OE, overexpression; Pias4, protein inhibitor of activated STAT 4; qPCR, quantitative PCR; RT-qPCR, quantitative reverse transcription PCR; siNC, siRNA against negative control; siRNA, small interfering RNA; SUMO2, small ubiquitin-like modifier; *Syce1*, synaptonemal complex central element protein 1; tdTomato, tandem dimeric Tomato; Zscan4d, zinc finger and SCAN domain containing 4D.

To figure out how Dppa2 activates zygotic transcriptional program, we decided to identify the downstream targets of Dppa2. We searched through a previously published chromatin immunoprecipitation sequencing (ChIP-seq) data of Dppa2 in mouse ESCs [40] and found no enrichment of Dppa2 binding on 2C genes globally. Interestingly, though, we found that the gene body of Dux was bound by Dppa2 (Fig 8A), raising the possibility that Dppa2 regulates zygotic transcriptional program through directly activating Dux. We then confirmed the binding of Dppa2 to Dux gene body by ChIP-qPCR (Fig 8B). Consistently, Dppa2 OE significantly increased the expression of Dux (Fig 8C). Moreover, knocking down Dux significantly inhibited the effects of Dppa2 OE in activating 2C-specific genes (Fig 8C) and increasing the fraction of 2C-like cells (Fig 8D). Taken together, these results suggest that Dppa2 activates zygotic transcriptional program through directly up-regulating Dux.

## Discussion

The transcription factor DUX resides at the top of a transcriptional hierarchy to activate zygotic transcriptional program during the 2C stage [10–12]. However, DUX itself is only beginning to be expressed at the early phase of ZGA; therefore, other transcriptional factors activating the transcription of Dux must exist. Here, we confirmed recent findings [15,16] that two related transcription factors Dppa2 and Dppa4 activate zygotic transcription program through directly activating Dux (Fig 8E). Both Dppa2 and Dppa4 are required for the activation of ZGA transcripts in 2C-like cells. Whereas Dppa2 OE sufficiently activates zygotic transcriptional program, Dppa4 OE is not sufficient and only enhances the function of Dppa2. In addition, we identified a Sumo2 E3 ligase Pias4, which inhibits the zygotic transcriptional program, likely through sumoylating multiple inhibitors and activators in regulating zygotic transcription. We further showed that the function of Pias4 is at least partially through sumoylating DPPA2, which turns DPPA2 from an activator to a potent inhibitor of zygotic transcriptional program. Consistent with its essential function as a brake for zygotic transcriptional program, the PIAS4 protein level was down-regulated at the early phase of ZGA and in 2C-like ESCs. However, the exact mechanism in regulating Pias4 expression is different between ZGA and 2C-like ESCs. Future investigation is needed to clarify this discrepancy. Furthermore, Pias4 OE or sumoylated DPPA2 inhibited the emergence of 2C-like cells and impaired early embryo development in mouse. Together, our study identifies key molecular rivals upstream of Dux in regulating the transition of ESCs into 2C-like cells and zygotic transcriptional program.

Sumo modification is involved in numerous cellular progresses, such as DNA damage response [25,26], signal transduction [41], and nuclear-cytosolic transport [42–44]. Increasing evidence shows that sumoylation functions in transcription regulation through modifying chromatin regulators [22–24] and transcription factors [20, 21]. In particular, SUMO was reported to repress the expression of ERVs such as MERVL through modification of Trim28, which is essential for the assembly of inhibitory ZFP809/TRIM28/SET domain, bifurcated 1 (SETDB1) machinery [17,24]. A recent study found that sumoylation acts as "glue" to recruit many chromatin regulators and transcription factors to safeguard cell state [45]. During the initial phase of induced pluripotent stem cell (iPSC) reprogramming, sumoylation acts on chromatin to prevent the inactivation of somatic enhancers by retaining somatic transcription factors. While in ESCs and iPSCs, sumoylation preserves the identity of stem cells by stabilizing repressors on heterochromatin dependent on trimethylation of histone H3 lysine 9 (H3K9me3), including Dux locus, to inhibit zygotic transcriptional program. The specific function of sumoylation in different conditions is enforced by specific Sumo E3 ligase [31]. However, which E3 ligase is responsible for regulating ESC identity was unknown. In our study, we identified Pias4 as the specific E3 ligase repressing the transition of ESCs into 2C-like cells. We noticed that Pias4 deletion leads to decreased viability perinatally [46,47], consistent with its important function during embryogenesis. However, the survived *Pias4−/−* animals are largely normal and fertile. Therefore, alternative pathways may exist to repress zygotic transcriptional program. After fertilization, the maternal Pias4 mRNA is decreased at the early phase of the 2C stage. In contrast, during 2C-like state transition in ESCs, Pias4 mRNA level remains unchanged, but protein is dramatically down-regulated. The exact mechanisms underlying the regulation of Pias4 expression in these two different circumstances warrant future investigation.

Dppa2 and Dppa4 are closely linked genes tandemly locating on the same chromosome in both mouse and humans [35]. They both contain a SAP domain that is known as DNA binding domain [48]. In addition, Dppa4 was reported to directly bind to chromatin, particularly transcriptionally active regions [49]. More recently, Dppa2 and Dppa4 were reported to be up-regulated during chemically induced reprogramming processes when 2C-like program is activated [50]. In another study, Dppa2 and Dppa4 were found as accelerators during reprogramming to pluripotency [51]. Mechanistically, they work as a heterodimer bound to chromatin to initiate global chromatin decompaction, which also happens to be an important event during 2C-like cell transition in ESCs and ZGA at the 2C stage [6,11,52]. These studies suggest that besides activating Dux expression, Dppa2 and Dppa4 may also facilitate chromatin remodeling to promote zygotic transcriptional program. Dppa2 and Dppa4 are expressed throughout the preimplantation development and in ESCs [35, 53], but Dux is only activated at the 2C stage and in a minor population of ESCs. This controversy strongly suggests that additional factors must exist to restrain the activating function of Dppa2/4. Our study suggests that sumoylation might be one of these restraining factors, which is released in 2C stage and 2C-like cells because of the down-regulation of E3 ligase Pias4. In addition, other coactivators may be required for Dppa2/4, and the expression or activity of these factors is restrictive in the 2C stage and 2C-like cells. Furthermore, the accessible chromatin state of *Dux* gene may be a prerequisite for Dppa2/4 to function. It is worth noting that Dppa2 and Dppa4 single or double knockout did not affect early embryogenesis [54,55], possibly because of maternally deposited products. Dppa2- and Dppa4-knockout mice died early after birth, preventing further evaluation of fertility of these mice. Therefore, maternal knockout of Dppa2 and Dppa4 is required to provide definite evidence on their function in ZGA. However, injection of a Dppa2 lacking SAP domain [36] or a sumoylated Dppa2 (this study) impaired preimplantation development. The dominant negative effect of mutant Dppa2 suggests that even though the redundant factors exist, they must share a similar working mechanism with Dppa2. Elucidating these mechanisms or identifying additional factors will contribute to the complete understanding of molecular mechanisms underlying ZGA control and may potentially lead to efficient means to produce a large quantity of 2C-like cells for research and therapeutic applications.

## Materials and methods

### Ethics statement

Mice were bred and maintained under specific pathogen–free (SPF) conditions in the institutional animal facilities at Peking University and Tsinghua University. All animal protocols were approved by Institutional Animal Care and Use Committees (IACUC) of Peking University (IMM-WangYM-1) and Tsinghua University (16-NJ-1), both of which are accredited by the Association for Assessment and Accreditation of Laboratory Animal Care International (AAALAC).

### Cell culture and construct of reporter cell lines

The ESC culture medium consisted of KnockOut DMEM (Gibco, Cat. # 10829081) with 15% FBS (Hyclone, Cat. # SH3007103), 1,000 U/ml mouse leukemia inhibitory factor (1,000 U/ml), 0.1 mM nonessential amino acids (Gibco, Cat. # 11140050), 0.1 mM β-mercaptoethanol, 1 mM L-glutamine (Gibco, Cat. # 25030081), and penicillin (100 U/ml) and streptomycin (100 μg/ml). For culture of ESC lines, the medium was changed daily, and cells were routinely passaged every other day. For generation of 2C::tdTomato reporter cell line, the MERVL–LTR–tdTomato reporter constructs made as previously described [5] were linearized and transfected into ESCs by electroporation. The cells were then selected with G418 for 7 d. Colonies containing tdTomato-positive cells were then picked and expanded. All cell lines were kept under constant drug selection with G418 to prevent transgene silencing. Mycoplasma detection tests were conducted routinely to ensure mycoplasma-free conditions throughout the study.

### RNA extraction and RT-qPCR

Total RNA was extracted following standard Trizol protocol (Invitrogen, Cat. # 15596026). For RT-qPCR analysis, complementary DNA (cDNA) were generated from 500 ng total RNA using HiScript II Q RT SuperMix for qPCR (Vazyme, Cat. # R223). qPCR was performed using AceQ qPCR SYBR Green Mater Mix (Vazyme, Cat. # Q141) in 96-well dishes in three biological replicates on StepOnePlus Real-Time PCR System (Applied Biosystems) with standard protocols. The expression levels were plotted relative to *b-actin*. Primers for qPCR are listed in S7 Table.

### CRISPR-Cas9 knockout and CRISPRi screening

To knock out Pias4, a single guide RNA (gRNA) sequence was designed by http://crispr.mit.edu/ to target Pias4 exon2. The gRNA sequence is 5′-GACAUGCUUGGUAACUAUGU-3′. Knockout of Pias4 was verified by genomic PCR followed by sequencing, RT-qPCR, and western blotting analysis.

For CRISPRi screening, a single gRNA was designed by http://crispr-era.stanford.edu/ for each candidate gene. The gRNA was cloned into a plasmid containing dCas9-krab. For screening, 0.05 million ESCs were plated in 12-well plate, and then 1 μg plasmid containing gRNA and dCas9-krab was transfected using lipofectamine 3000 (Invitrogen, Cat. # L3000) following the manufacturer's protocol. ESCs were selected with 300 μg/ml hygromycin 24 h post transfection for 4–5 d. Cells were then collected for flow cytometry analysis and RT-qPCR experiments.

### siRNA transfection

ESCs were transfected with siRNA (S8 Table) using DharmaFECT 1 (Dharmacon, Cat. # T-2001) reagent following the manufacturer’s instructions. NC is a siRNA sequence derived from *Caenorhabditis elegans* genome that does not target any mammalian genes, provided by GenePharma (Shanghai, China) as a control for siRNA transfection. Typically 2 days after transfection, cells were collected for flow cytometry, immunoblot, and RNA extraction.

### Immunoblots

Cells were collected and directly lysed in Mammalian Protein Extraction Reagent (Cwbiotech, Cat. # CW0889) with Protease Inhibitor Cocktail (100x, Cwbiotech, Cat. # CW0889). Proteins were quantified following manufacturer’s instructions using Pierce BCA Protein Assay Kit (Thermo Scientific). Equal amounts of proteins were loaded for immunoblotting. Antibodies used were rabbit anti-GAPDH (1:1,000, Bioworld Technology, Cat. # MB001), mouse anti-Flag (1:1,000, Sigma, Cat. # F3165), rabbit anti-HA (1:1,000 CST, Cat. # C29F4), rabbit anti-Pias4 (1:1,000, anti-PIAS4, Proteintech, Cat. # 14242-1-AP), and rabbit anti-Dppa2 (1:1,000, Abcam, Cat. # ab9138). Anti-rabbit and anti-mouse secondary antibodies were from LI-COR, and membranes were imaged using Odyssey. For anti-HA in Fig 5A and anti-Dppa2 in S4B and S4D Fig, HRP-conjugated anti-rabbit secondary antibodies were used and membranes were imaged using the Western ECL Substrate (Millipore, Cat. # WBKLS0500).

### Detection of sumoylated proteins

Cells were lysed in a solution containing 0.15 M Tris-HCl (pH 6.7), 5% SDS, and 30% glycerol, which was then diluted in 1:10 in PBS/0.5% Nonidet P-40 plus complete protease inhibitor. Anti-Flag monoclonal antibody (3 μg, Sigma, Cat. # F3165) was added to the lysate and incubated for 1 h at 4°C with gentle inversion mixing, after which 30 μl protein G Dynabeads (Invitrogen, Cat. # 10003) was added. After incubation for 3 h to overnight, the beads were collected and washed four times with ice-cold PBS, 0.5% Nonidet P-40 plus complete protease inhibitor mixture. Immunoprecipitated proteins were analyzed by SDS-PAGE and immunoblot using anti-HA monoclonal antibody (CST, Cat. # C29F4) and mouse anti-Flag (1:1,000, Sigma, Cat. # F3165).

### Sumoylation IP-MS

His6-Sumo2 pull-down was performed as previously described [56]. Briefly, cells were washed in PBS and lysed in lysis buffer (6 M guanidinium-HCl, 10 mM Tris, 100 mM sodium phosphate buffer [pH 8.0], 5 mM β-mercaptoethanol, 1 mM imidazole). Ni2+ NTA magnetic agarose beads (50 μl, Qiagen) were added to cell lysis and incubated at 4°C overnight. After incubation, the beads were washed once in lysis buffer, once in wash buffer (pH 8.0) (8 M urea, 10 mM Tris, 100 mM sodium phosphate buffer [pH 8.0], 0.1% Triton X-100, 5 mM β-mercaptoethanol), and three times in wash buffer (pH 6.3) (8 M urea, 10 mM Tris, 100 mM sodium phosphate buffer [pH 6.3], 0.1% Triton X-100, 5 mM β-mercaptoethanol, 10 mM imidazole). Sumoylated proteins were eluted from the beads using elution buffer (100 mM sodium phosphate buffer [pH 6.8], 200 mM imidazole) for MS analysis. Immunoprecipitated proteins were analyzed by SDS-PAGE. For LC-MS/MS analysis, the eluted peptides were sprayed into a Velos Pro Orbitrap Elite mass spectrometer (Thermo Scientific, USA) equipped with a nano-ESI source. The mass spectrometer was operated in data-dependent mode with a full MS scan in FT mode at a resolution of 120,000 followed by collision-induced dissociation (CID) MS/MS scans on the 15 most abundant ions in the initial MS scan. To identify Pias4 substrate proteins, we compared the count of distinct sequences that have significant scores (prot_sequences_sig) for each protein in siNC versus si-Pias4 samples. Candidate proteins selected for further CRISPRi screening were chosen based on the following criteria: The candidate has to be detected in at least two of four siNC samples; the average #PSM for a candidate must be >1 in siNC samples; the ratio of average #PSM of siPias4 versus siNC is ≥1.25-fold at the 48-hr or 72-hr time point. In total, 97 candidates passed these criteria. For screening, housekeeping genes Actb and Ctc1 were not included; Mtco2 and Tmlhe were not included, as no appropriate CRISPRi gRNA can be designed based on their sequences; Sumo1 was not included, as we already showed that knocking down Sumo1 decreased the expression of Dux and other 2C-like genes (S1B Fig).

### Flow cytometry

Cells were trypsinized and resuspended in ice-cold PBS containing 2% FBS. Sorting was performed using a BD FACSAria III. During sorting, cells were collected in culture medium and kept at 4°C. Quantifying the population of 2C::tdTomato-positive cells was performed by BD LSRFortessa SORP. Data were analyzed using FlowJo software.

### In vitro transcription

The cDNA encoding the desired genes was amplified and cloned under the control of a T7 promoter. After linearization by a restriction enzyme PmeI, the construct was purified with phenol-chloroform extraction and ethanol precipitation. mRNA was synthesized by in vitro transcription using a HiScribe T7 ARCA mRNA Kit (NEB, Cat. # E2065) according to the manufacturer’s instructions. The synthesized mRNA was purified by lithium chloride precipitation and diluted with nuclease-free water. mRNA aliquots were stored at −80°C until use.

### Microinjection

For mouse embryo collection, 4- to 6-week-old C57BL/6N female mice (Vital River) were injected with PMSG (10 IU) and then with hCG (10 IU) 48 h later. Female mice were then mated with C57BL/6N male mice. Zygotes were collected and kept in KSOM medium (Millipore, Cat. # MR-020P-5F) pregassed under 5% CO_2_ at 37°C. Embryos were then transferred to M2 medium (Millipore, Cat. # MR-015-D) and microinjected with in vitro transcribed mRNA using a FemtoJet device. The mRNA concentration of GFP–Pias4 and GFP–Pias4 W356A was 200 ng/μl, and control GFP–NLS was 100 ng/μl. The mRNA concentration for GFP–t2a–Dppa2 and GFP–t2a–Sumo2–Dppa2 was 500 ng/μl; control GFP was 100 ng/μl. Concentrations of control GFP plasmids were determined to make sure that approximately equivalent amount of GFP protein was expressed for each group. For each group, 6 embryos were collected per stage of 1-cell, early 2C, late 2C, 4-cell, and 8-cell embryos at 20 h, 32 h, 50 h, 64 h, and 74 h post hCG, respectively, for qPCR analysis.

### Chimeric blastocyst assay

The zona pellucida of 8 cell–stage embryos were removed by a short exposure to acidic Tyrode’s solution (Sigma, Cat. # T1788). The denuded embryos were placed into each concaved microwell created by a smooth depression using the aggregation needle. Four ESCs labeled by mRuby2 fluorescence were transferred into each concaved microwell and cocultured with the denuded embryo overnight.

### Single-cell and single-embryo RT-qPCR

Single cell or single embryo was transferred into 2 μl mild hypotonic lysis buffer composed of 0.2% Triton X-100 and 2 U/μl of RNase inhibitor (Ambion, Cat. # AM2684) and 10 fg spike-in GFP or tagRFP mRNA. The single-cell lysates were mixed with 1 μl of anchored oligo-dT primer (10 μM) and 1 μl of dNTP mix (10 μM, Invitrogen, Cat. # 18427), denatured at 72°C for 3 min. The first-strand reaction mix, containing 0.5 μl SuperScript II reverse transcriptase (200 U/μl, Invitrogen, Cat. # 18064), RNase inhibitor (40 U/μl, Ambion, Cat. # AM2684), 2 μl Superscript II First-Strand Buffer (5x, Invitrogen, Cat. # 18064), 0.25 μl DTT (100 mM, Invitrogen, Cat. # 18064), 2 μl betaine (5 M, Sigma, Cat. # 61962), 0.06 μl MgCl2 (100 mM, Sigma, Cat. # 1374248), 0.1 μl TSO (100 μM), and 0.29 μl nuclease-free water, was added to each sample. Reverse transcription reaction was carried out by incubating at 42°C for 90 min, followed by 10 cycles of 50°C for 2 min and 42°C for 2 min). Finally, the reverse transcriptase was inactivated by incubation at 70°C for 15 min. For PCR pre-amplification, the PCR mater mix containing 12.5 μl KAPA HiFi HotStart ReadyMix (2x, KAPA Biosystems, Cat. # KK602), 0.25 μl ISPCR primers (10 μM), and 2.25 μl nuclease-free water was added to each sample. The program was carried out by incubating at 98°C for 30 min, followed by 18 cycles of 98°C for 20 s, 67°C for 15 s, and 72°C for 6 min, with a final extension at 72°C for 5 min. PCR products were purified using a 1:1 ratio of VAHTS DNA Clean Beads (Vazyme, Cat. # N411), with the final elution performed in 20 μl of nuclease-free water. The qPCR was performed using AceQ qPCR SYBR Green Mater Mix (Vazyme, Cat. # Q141). Oligo-dT primer: 5′-AAGCAGTGGTATCAACGCAGAGTACT30VN-3′; TSO: 5′-AAGCAGTGGTATCAACGCAGAGTACATrGrG+G-3′; ISPCR oligo: 5′-AAGCAGTGGTATCAACGCAGAGT-3′. For single-embryo qPCR data analysis, the spike-in mRNA was used as a control, and data were normalized to zygote stage.

### IF staining and PLA

Cells were fixed with 4% paraformaldehyde for 20 min at room temperature. After the fixation, cells were permeabilized with 0.25% Triton X-100 for 20 min at room temperature and blocked with 3% FBS in PBS for 1 h at room temperature. Cells were then incubated with primary antibodies (1:100, anti-PIAS4, Proteintech, Cat. # 14242-1-AP) diluted in PBS with 3% FBS for 2 h. After washing three times in PBS, the cells were incubated with secondary antibody (1:200, anti-rabbit IgG Alexa fluor 488) for 1 h and followed by DAPI staining. For preimplantation embryos’ IF staining, 0.1% Tween-20 was added in PBS. For PLA, the permeabilized cells were incubated in PBS containing two primary antibodies (1:700, anti-Dppa2, Abcam, Cat. # ab91318; 1:500, anti-Sumo2, Cytoskeleton, Cat. # ASM23) followed by Duolink in situ PLA (Sigma-Aldrich) anti-mouse (minus) and anti-rabbit (plus) probes and detection reagents Green according to manufacturer’s instructions.

### RNA-seq and bioinformatics analysis

Total RNA was enriched twice with poly-T oligo-attached magnetic beads and then subjected to the synthesis of double-stranded (ds) cDNA. The ds-cDNA was ligated to adaptors from NEB and sequenced by Illumina Genome Analyzer (Novogene, Tianjin, China). Sequencing reads were aligned to the mouse genome (mm10) with STAR (version 2.5.0) using the GENCODE transcript annotation as transcriptome guide. All programs were processed following default settings except for special annotation. The FPKM value generated by Cufflinks was used to quantify the expression level. The enrichment of selected gene sets was calculated by java GSEA Desktop Application. R 3.1.1 and Matlab were used for the generation of scatter plot and boxplot. The 2C-specific ZGA genes are genes activated during ZGA (the 2C stage) that are also enriched in 2C::tdTomato^+^ cells from [5]. The Dux-induced gene set was from [11]. The list of MERVL–LTR-driven transcripts was from [6]. The list of genes induced by mir-34a knockout was from [13]. The list of genes induced by G9a knockout was from [5]. The list of genes induced by LINE1 knocking down was from [14]. The lists of genes induced by p150 or p60 knocking down were from [8].

### ChIP-qPCR

Cells were fixed for 15 min at room temperature in culture medium with formaldehyde (0.9% final concentration). Formaldehyde was then quenched with glycine (125 mM final). Cells were washed twice with cold PBS. Lift cells by treating cells with 0.25% trypsin for 10 min. Spin down cells and wash with cold PBS. Resuspend the cell with 2x lysis buffer (50 mM Tris-HCl [pH 8.1], 10 mM EDTA, 1% SDS with fresh protease inhibitor, PMSF, and DTT) for 10 min on ice. Chromatin extracts containing DNA fragments with an average size of 200–500 bps were immunoprecipitated using IgG or anti-FLAG (Sigma, Cat. # F3165) overnight at 4°C. Immunoprecipitated complexes were successively washed with low-salt buffer, high-salt buffer, LiCl buffer, and TE buffer and eluted with TE added with proteinase K. The reverse cross-link was performed by incubation of the samples overnight at 65°C. After reverse cross-linking, DNA was purified using TIANquick Midi Purification Kit (TIANGEN) according to the manufacturer’s instructions. Primer sequences used in ChIP assay are listed in S9 Table.

### Quality control of sequencing data

The quality of every library was determined using the fastqc tool (http://www.bioinformatics.babraham.ac.uk/projects/fastqc/). Reads were subsequently trimmed and adapters clipped using the fastq-mcf (https://github.com/ExpressionAnalysis/ea-utils/blob/wiki/FastqMcf.md). Only reads with none of the known high-throughput sequencing adapters, longer than 25 bps, with a mean quality score above 30 and maximum 1 N-call were kept.

### Quantification and statistical analysis

The number of independent experimental replications, the definition of center, and precision measures are reported in the figure legends (*n*, mean ± SD). *p* < 0.05 is generally considered as statistically significant. Statistical analyses were performed using the GraphPad Prism v6 software. Statistical significance was assessed by two-tailed *t* test except when specified in the figure legends. For boxplots, upper and lower whiskers are defined as respectively Q3 + 1.5 × IQR and Q1 − 1.5 × IQR, with Q1 and Q3 being the first and third quartile of the plotted distribution and IQR the interquartile range; the *p*-value was determined by Wilcoxon signed rank test. For multiple comparison, the *p*-value was calculated by one-way or two-way ANOVA followed with Dunnett's test.

## Supporting information

S1 Fig Sumo2 and Sumo E3 ligase Pias4 repress zygotic transcriptional program. (A) Flow cytometry analysis of 2C::tdTomato-positive cells in Sumo1- and Sumo2-knockdown ESCs. (B) RT-qPCR of Dux and other 2C-specific genes in Sumo1- and Sumo2-knockdown ESCs. The β-actin gene was used as a control. For each gene, data were normalized to the mRNA level of ESCs transfected with control siRNAs. Shown are mean ± SD, *n* = 3. The *p*-value was calculated by one-way ANOVA followed by two-tailed Dunnett's test. (C) Flow cytometry analysis of 2C::tdTomato-positive cells in ESCs transfected with siRNAs against various Sumo E3 ligases. (D) Box-and-whisker plots showing expression of genes up-regulated by mir-34a knockout, G9a knockout, LINE1 knockdown, and Caf-1 p150 or p60 subunit knockdown in cells transfected with Pias4 siRNA. The *p*-value was determined by Wilcoxon signed rank test. (E) Assays confirming knockout of *Pias4*. Western blotting analysis of PIAS4 protein in wild-type and *Pias4−/−* ESCs (left). Immunofluorescence staining of PIAS4 protein in wild-type and *Pias4−/−* ESCs. Scale bar, 50 μm (right). (F) Fraction of 2C::tdTomato-positive cells in wild-type and *Pias4−/−* ESCs. Shown are representative images for different colonies. Scale bars, 100 μm. Source data for B can be found in the supplemental data file (S1 Data). 2C, 2-cell; Caf-1, chromatin assembly factor; Dux, double homeobox; ESC, embryonic stem cell; LINE1, long interspersed nuclear element; mir-34a, microRNA 34a; Pias4, protein inhibitor of activated STAT 4; RT-qPCR, quantitative reverse transcription PCR; siRNA, small interfering RNA; Sumo, small ubiquitin-like modifier; tdTomato, tandem dimeric Tomato.
(TIF)

S2 Fig PIAS4 is down-regulated in 2C-like cells and 2C stage during zygotic genome activation. (A) Expression of Pias4 in 2C-like cells. RNA-seq data from [8]. (B) Single-embryo RT-qPCR of Dux, MERVL, and Zscan4d in preimplantation mouse embryos. Spike-in GFP mRNA was used as a control. Data were normalized to zygote. Red bars indicate mean. *n* = 4–6. Each dot represents one embryo. The *p*-value was as indicated, two-tailed Student’s *t* test. Source data for A and B can be found in the supplemental data file (S1 Data). Dux, double homeobox; GFP, green fluorescent protein; MERVL, murine endogenous retrovirus-L; Pias4, protein inhibitor of activated STAT 4; RNA-seq, RNA sequencing; RT-qPCR, quantitative reverse transcription PCR; Zscan4d, zinc figure and SCAN domain containing 4D.
(TIF)

S3 Fig Identification of Pias4 substrates that regulate zygotic transcriptional program. (A) Average peptide counts of proteins in 6xHis-Sumo2 pull-down/MS for ESCs treated with siRNAs against NC or Pias4. Proteins represented by black dots or colored dots were selected as candidate genes for CRISPRi screening. (B) Gene Ontology analysis of Pias4 substrates identified by Sumo2 IP. (C) Fold change of the percentage of Zscan4::GFP-positive cells in Pias4 substrate CRISPRi ESCs transfected with siNC or si-Pias4. Each dot represents an ESC line transfected with CRISPRi constructs targeting a candidate Pias4 substrate protein. The red line indicate the value 1.0. To calculate the fold change, the fraction of Zscan4::GFP-positive cells in each samples is divided by the fraction of Zscan4::GFP-positive cells in control CRISPRi ESCs. (D) Fold change of the percentage of 2C::tdTomato-positive cells in ESCs transfected with siRNAs against various Pias4 substrates in the presence of siNC or si-Pias4. Black bars indicate mean. *n* = 3–4. To calculate the fold change, the fraction of MERVL::tdTomato-positive cells in ESCs treated with different siRNAs is divided by the fraction of MERVL::tdTomato-positive cells ESCs treated with siNC. Source data for C and D can be found in the supplemental data file (S1 Data). CRISPRi, clustered regularly interspaced short palindromic repeat interference; ESC, embryonic stem cell; GFP, green fluorescent protein; IP, immunoprecipitation; MERVL, murine endogenous retrovirus-L; MS, mass spectrometry; NC, negative control; Pias4, protein inhibitor of activated STAT 4; siRNA, small interfering RNA; Sumo2, small ubiquitin-like modifier; tdTomato, tandem dimeric Tomato; Zscan4, zinc finger and SCAN domain containing 4.
(TIF)

S4 Fig Dppa2 and Dppa4 are essential for the activation of zygotic genome activation. (A) RT-qPCR of Pias4 and Dppa2 (left), Dux, and other 2C-specific genes (right) in ESCs treated with siRNAs against Pias4 and Dppa2 individually or in combination. The β-actin gene was used as a control. For each gene, data were normalized to the mRNA level of wild-type ESCs. Shown are mean ± SD, *n* = 3. The *p*-value was calculated by two-way ANOVA followed by two-tailed Dunnett's test. (B) RT-qPCR of Dppa2 and Dppa4 (left), Dux, and other 2C-specific genes (right) in ESCs treated with siRNAs against Dppa2 and Dppa4 individually or in combination. The β-actin gene was used as a control. For each gene, data were normalized to the mRNA level of wild-type ESCs. Shown are mean ± SD, *n* = 3. The *p*-value was calculated by two-way ANOVA followed by two-tailed Dunnett's test. Source data for A and B can be found in the supplemental data file (S1 Data). 2C, 2-cell; Dppa, developmental pluripotency associated; Dux, double homeobox; ESC, embryonic stem cell; Pias4, protein inhibitor of activated STAT 4; RT-qPCR, quantitative reverse transcription PCR; siRNA, small interfering RNA.
(TIF)

S5 Fig Dppa4 is essential for the activation of zygotic transcriptional program. (A) GSEA for 2C-specific genes in ESCs transfected with control siRNAs or siRNAs against Dppa4. For the x-axis, genes were ranked based on the ratio of siNC versus si-Dppa2 ESCs. (B) MA plots showing gene expression changes in Dppa4-knockdown ESCs. Red dots indicate Dux-induced genes. Out of 123 Dux-induced genes, 77 were down-regulated in Dppa4-knockdown ESCs. Fold enrichment and *p*-value are shown. The *p*-value was calculated by hypergeometric test. (C) MA plots showing gene expression changes in Dppa4-knockdown ESCs. Red dots indicate MERVL–LTR-driven genes. Out of 161 MERVL–LTR-driven genes, 72 were down-regulated in Dppa4-knockdown ESCs. Fold enrichment and *p*-value are shown. The *p*-value was calculated by hypergeometric test. (D) Expression of genes down-regulated in Dppa4-knockdown ESCs in preimplantation mouse embryos. Center line, median; box limits, upper and lower quartiles; whiskers, 1.5× interquartile range. Preimplantation RNA-Seq data are from [32]. 2C, 2-cell; Dppa, developmental pluripotency associated; Dux, double homeobox; ESC, embryonic stem cell; GSEA, gene set enrichment analysis; LTR, long terminal repeat; MERVL, murine endogenous retrovirus-L; RNA-seq, RNA sequencing; siNC, siRNA against negative control; siRNA, small interfering RNA.
(TIF)

S6 Fig Gene expression changes were largely similar in ESCs knocking down Dppa2 and Dppa4 individually or in combination. (A) Heatmap showing gene expression changes in ESCs transfected with control siRNAs and siRNAs against Dppa2 and Dppa4 individually or in combination. Only differentially expressed genes are shown. For each gene, data were normalized to the average of four samples. Color key is shown right (Log2). (B) Box-and-whisker plots showing expression of genes up-regulated by mir-34a knockout, G9a knockout, LINE1 knockdown, and Caf-1 p150 or p60 subunit knocked down in cells transfected with Dppa2 siRNAs or Dppa4 siRNAs separately or in combination. The *p*-value was determined by Wilcoxon signed rank test. Caf-1, chromatin assembly factor; Dppa, developmental pluripotency associated; ESC, embryonic stem cell; LINE1, long interspersed nuclear element; mir-34a, microRNA 34a; siRNA, small interfering RNA.
(TIF)

S7 Fig SUMO2–DPPA2 is lower in MERVL-positive cells. (A) RT-qPCR using Dppa2 primers in Sumo2ΔGG–Dppa2 overexpressing ESCs. The β-actin gene was used as a control. Data were normalized to the mRNA level of control ESCs. Shown are mean ± SD, *n* = 3. (B) Western blotting analysis of sumoylated and nonsumoylated Dppa2 in control and Sumo2ΔGG–Dppa2-overexpressing ESCs. This ESC colony was used for experiments in Fig 6B–6D. Left, representative gel blot images; right, relative ratio of Sumo2ΔGG–Dppa2 versus nonsumoylated Dppa2 in Sumo2ΔGG–Dppa2-overexpressing ESCs. Shown are mean ± SD, *n* = 3. The *p*-value was calculated by two-tailed Student's *t* test. (C) Flow cytometry analysis of control and Sumo2ΔGG–Dppa2-overexpressing ESCs treated with NC and Pias4 siRNAs. Shown are presented as mean ± SD, *n* = 3. The *p*-value was calculated by two-tailed Student’s *t* test. (D) Western blotting analysis of sumoylated and nonsumoylated Dppa2 in control and various Sumo2ΔGG–Dppa2-overexpressing ESCs. The ratio of sumoylated versus nonsumoylated Dppa2 is shown at the bottom of the gel. The colony #5 is picked for experiments in E and F. (E) RT-qPCR of Dux and other 2C-specific genes in control and #5 Sumo2ΔGG–Dppa2-overexpressing ESCs. The β-actin gene was used as a control. For each gene, data were normalized to the mRNA level of wild-type ESCs. Shown are mean ± SD, *n* = 3. The *p*-value was calculated by two-tailed Student’s *t* test. (F) Fraction of 2C::tdTomato-positive cells in control and #5 Sumo2ΔGG–Dppa2-overexpressing ESCs. Data are presented as mean ± SD, *n* = 3. The *p*-value was calculated by two-tailed Student’s *t* test. (G) PLA assay of SUMO2 and DPPA2 in 2C::tdTomato-positive and negative ESCs. Shown are representative images (left) and quantification of number of PLA dots/nucleus with mean ± SD (right). Scale bars, 10 µm. *n* = 42 tdTomato-positive cells and 194 tdTomato-negative cells. Each dot represents one nucleus. (H) IF staining of DPPA2 protein in MERVL::tdTomato-positive and negative ESCs. Shown are representative images (left) and quantification of fluorescence intensity for each cell with mean ± SD (right). Scale bars, 10 μm. *n* = 65 tdTomato-positive cells and 700 tdTomato-negative cells. Each dot represents one cell. The *p*-value is calculated by two-tailed Student's *t* test. (I) DPPA2 localization in MERVL::tdTomato-positive and negative ESCs. Shown are representative images for IF staining of DPPA2 protein. Arrowhead points to 2C::tdTomato-positive ESCs. Scale bars, 5 μm. Source data for A-C and E-H can be found in the supplemental data file (S1 Data). 2C, 2-cell; Dppa2, developmental pluripotency associated 2; Dux, double homeobox; ESC, embryonic stem cell; IF, immunofluorescence; MERVL, murine endogenous retrovirus-L; NC, negative control; Pias4, protein inhibitor of activated STAT 4; PLA, proximity ligation assay; siRNA, small interfering RNA; Sumo2, small ubiquitin-like modifier 2; tdTomato, tandem dimeric Tomato.
(TIF)

S8 Fig Overexpression of Dppa2 activates zygotic transcriptional program and Dppa4 enhances the function of Dppa2. (A) Fraction of 2C::tdTomato-positive cells in control and Dppa2-overexpressing ESCs treated with Sumo2 or Pias4 siRNAs. Data are presented as mean ± SD, *n* = 3. The *p*-value was calculated by two-tailed Student’s *t* test. (B) Box-and-whisker plots showing expression of genes up-regulated by mir-34a KO, G9a KO, LINE1 knockdown, and Caf-1 p150 or p60 subunit knockdown in cells overexpressing Dppa2. The *p*-value was determined by Wilcoxon signed rank test. (C) PCA mapped scatter plot: global protein coding genes (left) and repeat elements (right). Data for G9a KO from [5], P150 and P60 knockdown and 2C::EGFP+ cells from [8], Dux-overexpressing cells from [11], and LINE1-knockdown cells from [14]. Data were normalized to the control cell line of each study to exclude batch effects before PCA processing. (D) RT-qPCR of Dux and other 2C-specific genes in control, Dppa2-, Dppa4-, or Dppa2/4-overexpressing ESCs. The β-actin gene was used as a control. For each gene, data were normalized to the mRNA level of control ESCs. Shown are mean ± SD, *n* = 3. The *p*-value was calculated by one-way ANOVA followed by two-tailed Dunnett's test. (E) Fraction of 2C::tdTomato-positive cells in control, Dppa2-, Dppa4-, or Dppa2/4-overexpressing ESCs. Data are presented as mean ± SD, *n* = 3. The *p*-value was calculated by two-tailed Student’s *t* test. Source data for A, D, and E can be found in the supplemental data file (S1 Data). 2C, 2-cell; Dppa, developmental pluripotency associated; Dux, double homeobox; EGFP, enhanced green fluorescent protein; ESC, embryonic stem cell; KO, knockout; LINE1, long interspersed nuclear element; mir-34a, microRNA 34a; PCA, principle component analysis; Pias4, protein inhibitor of activated STAT 4; RT-qPCR, quantitative reverse transcription PCR; siRNA, small interfering RNA; Sumo2, small ubiquitin-like modifier 2; tdTomato; tandem dimeric Tomato.
(TIF)

S1 Table FPKM of genes in Pias4-siRNA-seq. List of gene expression in ESCs after Pias4 RNAi. Shown is the average of two replicates. ESC, embryonic stem cell; FPKM, fragments per kilobase of transcript per million fragments mapped; Pias4, protein inhibitor of activated STAT 4; RNAi, RNA interference; RNA-seq, RNA sequencing; siRNA, small interfering RNA.
(XLSX)

S2 Table FPKM of repeats in Pias4-siRNA-seq. List of repeat elements expression in ESCs after Pias4 RNAi. Shown is the average of two replicates. RNA-seq, RNA sequencing. ESC, embryonic stem cell; FPKM, fragments per kilobase of transcript per million fragments mapped; Pias4, protein inhibitor of activated STAT 4; RNAi, RNA interference; RNA-seq, RNA sequencing; siRNA, small interfering RNA.
(XLSX)

S3 Table Average #PSM of proteins identified in Sumo2 IP. List and #PSM of proteins identified in His6-SUMO2 IP-MS experiment. Candidates selected in CRISPRi screening are shaded in blue. CRISPRi, clustered randomly interspaced short palindromic repeat interference; IP, immunoprecipitation; MS, mass spectrometry; PSM, peptide spectrum match; SUMO2, small ubiquitin-like modifier 2.
(XLSX)

S4 Table FPKM of genes in Dppa2, Dppa4, and Dppa2/4 siRNA-seq. List of gene expression in ESCs after Dppa2, Dppa4, and Dppa2/4 RNAi. Shown is the average of two replicates. Dppa, developmental pluripotency associated; ESC, embryonic stem cell; FPKM, fragments per kilobase of transcript per million fragments mapped; RNAi, RNA interference; RNA-seq, RNA sequencing; siRNA, small interfering RNA.
(XLSX)

S5 Table FPKM of genes in Dppa2-OE RNA-seq. List of gene expression in ESCs overexpressing Dppa2. Shown is the average of two replicates. Dppa2, developmental pluripotency associated 2; ESC, embryonic stem cell; FPKM, fragments per kilobase of transcript per million fragments mapped; OE, overexpression; RNA-seq, RNA sequencing.
(XLSX)

S6 Table FPKM of repeats in Dppa2-OE RNA-seq. List of repeat elements expression in ESCs overexpressing Dppa2. Shown is the average of two replicates. Dppa2, developmental pluripotency associated 2; ESC, embryonic stem cell; FPKM, fragments per kilobase of transcript per million fragments mapped; OE, overexpression; RNA-seq, RNA sequencing.
(XLSX)

S7 Table Sequences for real-time qPCR primers. Sequence information of forward and reverse primers used for Pias4, Dppa2, Dppa4, and various ZGA transcripts in qPCR. Dppa, developmental pluripotency associated; qPCR, quantitative PCR; Pias4, protein inhibitor of activated STAT 4; ZGA, zygotic genome activation.
(XLSX)

S8 Table Sequences for siRNAs. Sequence information of sense siRNA strand for knocking down Pias4, Dppa2, Dppa4, Sumo1, Sumo2, various Sumo E3 ligases, and candidate ZGA regulators. Dppa, developmental pluripotency associated; Pias4, protein inhibitor of activated STAT 4; siRNA, small interfering RNA; Sumo, small ubiquitin-like modifier; ZGA, zygotic genome activation.
(XLSX)

S9 Table Sequences of ChIP-qPCR primers. Sequence information of forward and reverse primers for ChIP-qPCR analysis of various positions in *Dux* promoter and gene body. ChIP-qPCR, chromatin immunoprecipitation followed by quantitative polymerase chain reaction. ChIP-qPCR, chromatin immunoprecipitation–quantitative PCR; *Dux*, double homeobox.
(XLSX)

S1 Data Excel spreadsheet containing, in separate sheets, the underlying numerical data and data analysis for figure panels. Source data for main and supplemental figures in separate sheets. Mean, SD, and *p*-value are shown where applicable.
(XLSX)

## Abbreviations

2C: 2-cell
a.u.: arbitrary units
BFP: blue fluorescent protein
CAF-1: chromatin assembly factor-1
Cas9: CRISPR-associated protein 9
ChIP-seq: chromatin immunoprecipitation sequencing
CRISPR: clustered regularly interspaced short palindromic repeat
CRISPRi: CRISPR interference
Ct: cycle threshold
Dppa: developmental pluripotency associated
Dux: double homeobox
EP400: E1A binding protein p400
ERV: endogenous retrovirus
ESC: embryonic stem cell
FC: fold change
GFP: green fluorescent protein
GO: Gene Ontology
GV: germinal vesicle
H3K9me3: trimethylation of histone H3 lysine 9
HA: hemagglutinin
HEK293T: human embryonic kidney 293T
ICM: inner cell mass
IF: immunofluorescence
iPSC: induced pluripotent stem cell
LINE1: long interspersed nuclear element
Lsd1/Kdm1a: lysine (K)-specific demethylase 1A
LTR: long terminal repeat
MERVL: murine ERV-L
miR-34a: microRNA 34a
Mrpl38: mitochondrial ribosomal protein L38
MS: mass spectrometry
Nacc1: nucleus accumbens associated 1
NES: normalized enrichment score
OE: overexpression
PIAS4: protein inhibitor of activated STAT 4
PLA: proximity ligation assay
Prmt5: protein arginine N-methyltransferase 5
qPCR: quantitative PCR
RNA-seq: RNA sequencing
RPKM: reads per kilobase per million mapped reads
RT-qPCR: reverse transcription qPCR
SETDB1: SET domain, bifurcated 1
siNC: siRNA against negative control
siRNA: small interfering RNA
SSC-H: side scatter height
SUMO: small ubiquitin-like modifier
Syce1: synaptonemal complex central element protein 1
Tcstv: 2C-stage variable group
Tdh: L-threonine dehydrogenase
tdTomato: tandem dimeric Tomato
TE: trophectoderm
Trim28: tripartite motif-containing 28
Zfp: zinc finger protein
ZGA: zygotic genome activation
Zscan4: zinc finger and SCAN domain containing 4
