# Correction: DPPA2/4 and SUMO E3 ligase PIAS4 opposingly regulate zygotic transcriptional program

**DOI:** 10.1371/journal.pbio.3002379

**Published:** 2023-11-09

**Authors:** Yuelong Yan, Chao Zhang, Jing Hao, Xue-Lian Wang, Jia Ming, Li Mi, Jie Na, Xinli Hu, Yangming Wang

The first author’s name is spelled incorrectly. The correct name is: Yuelong Yan. The correct citation is: Yan Y, Zhang C, Hao J, Wang X-L, Ming J, Mi L, et al. (2019) DPPA2/4 and SUMO E3 ligase PIAS4 opposingly regulate zygotic transcriptional program. PLoS Biol 17(6): e3000324. https://doi.org/10.1371/journal.pbio.3000324
