# Supplementary figures and images for: DPPA2/4 and SUMO E3 ligase PIAS4 opposingly regulate zygotic transcriptional program
Source: PLoS Biol. 2019 Jun 21;17(6):e3000324. doi: 10.1371/journal.pbio.3000324 (PMC6608977; doi:10.1371/journal.pbio.3000324)

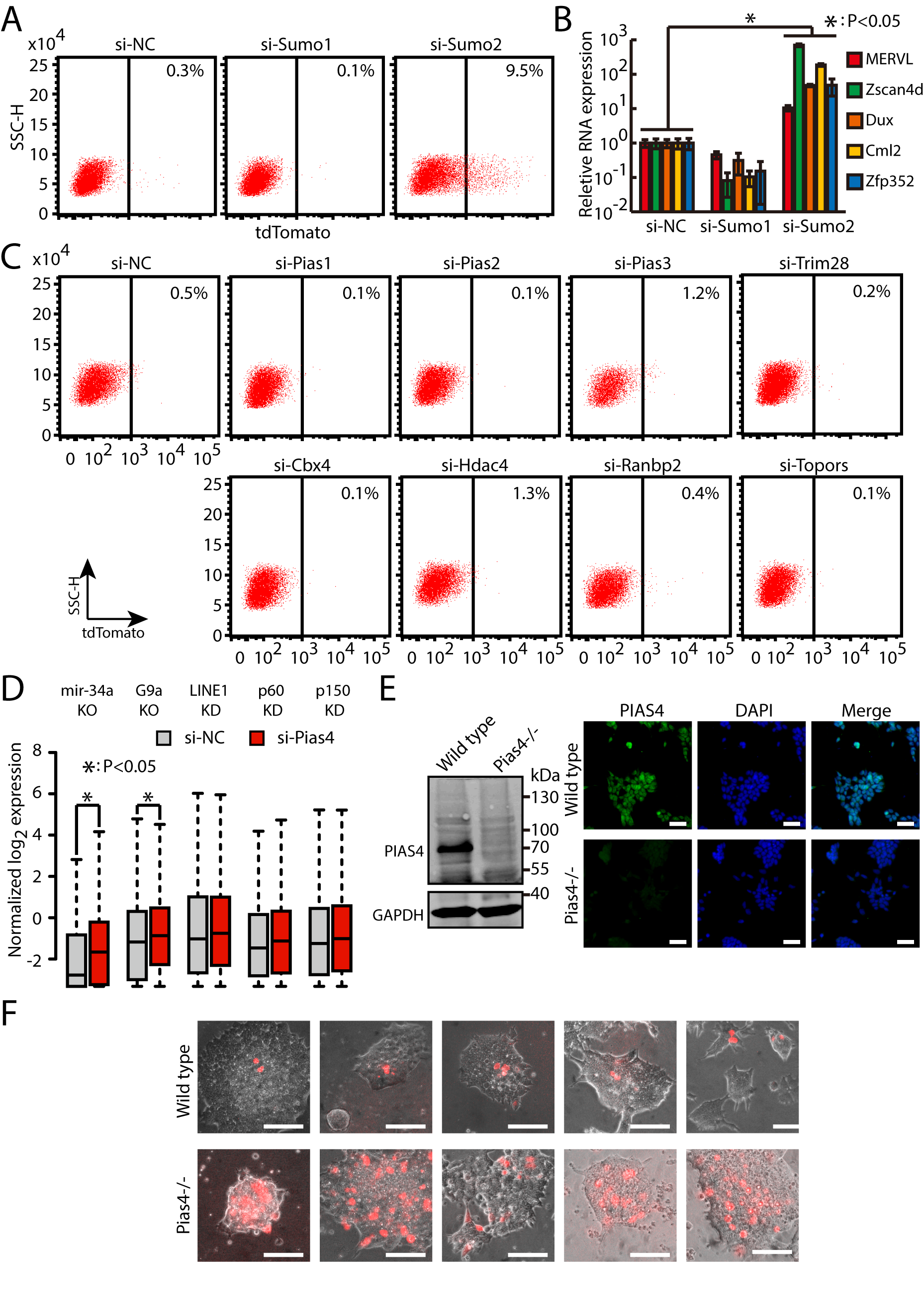

Supplement: S1 Fig — (A) Flow cytometry analysis of 2C::tdTomato-positive cells in Sumo1- and Sumo2-knockdown ESCs. (B) RT-qPCR of Dux and other 2C-specific genes in Sumo1- and Sumo2-knockdown ESCs. The β-actin gene was used as a control. For each gene, data were normalized to the mRNA level of ESCs transfected with control siRNAs. Shown are mean ± SD, n = 3. The p-value was calculated by one-way ANOVA followed by two-tailed Dunnett's test. (C) Flow cytometry analysis of 2C::tdTomato-positive cells in ESCs transfected with siRNAs against various Sumo E3 ligases. (D) Box-and-whisker plots showing expression of genes up-regulated by mir-34a knockout, G9a knockout, LINE1 knockdown, and Caf-1 p150 or p60 subunit knockdown in cells transfected with Pias4 siRNA. The p-value was determined by Wilcoxon signed rank test. (E) Assays confirming knockout of Pias4. Western blotting analysis of PIAS4 protein in wild-type and Pias4−/− ESCs (left). Immunofluorescence staining of PIAS4 protein in wild-type and Pias4−/− ESCs. Scale bar, 50 μm (right). (F) Fraction of 2C::tdTomato-positive cells in wild-type and Pias4−/− ESCs. Shown are representative images for different colonies. Scale bars, 100 μm. Source data for B can be found in the supplemental data file (S1 Data). 2C, 2-cell; Caf-1, chromatin assembly factor; Dux, double homeobox; ESC, embryonic stem cell; LINE1, long interspersed nuclear element; mir-34a, microRNA 34a; Pias4, protein inhibitor of activated STAT 4; RT-qPCR, quantitative reverse transcription PCR; siRNA, small interfering RNA; Sumo, small ubiquitin-like modifier; tdTomato, tandem dimeric Tomato. (TIF) [file pbio.3000324.s001.tif]

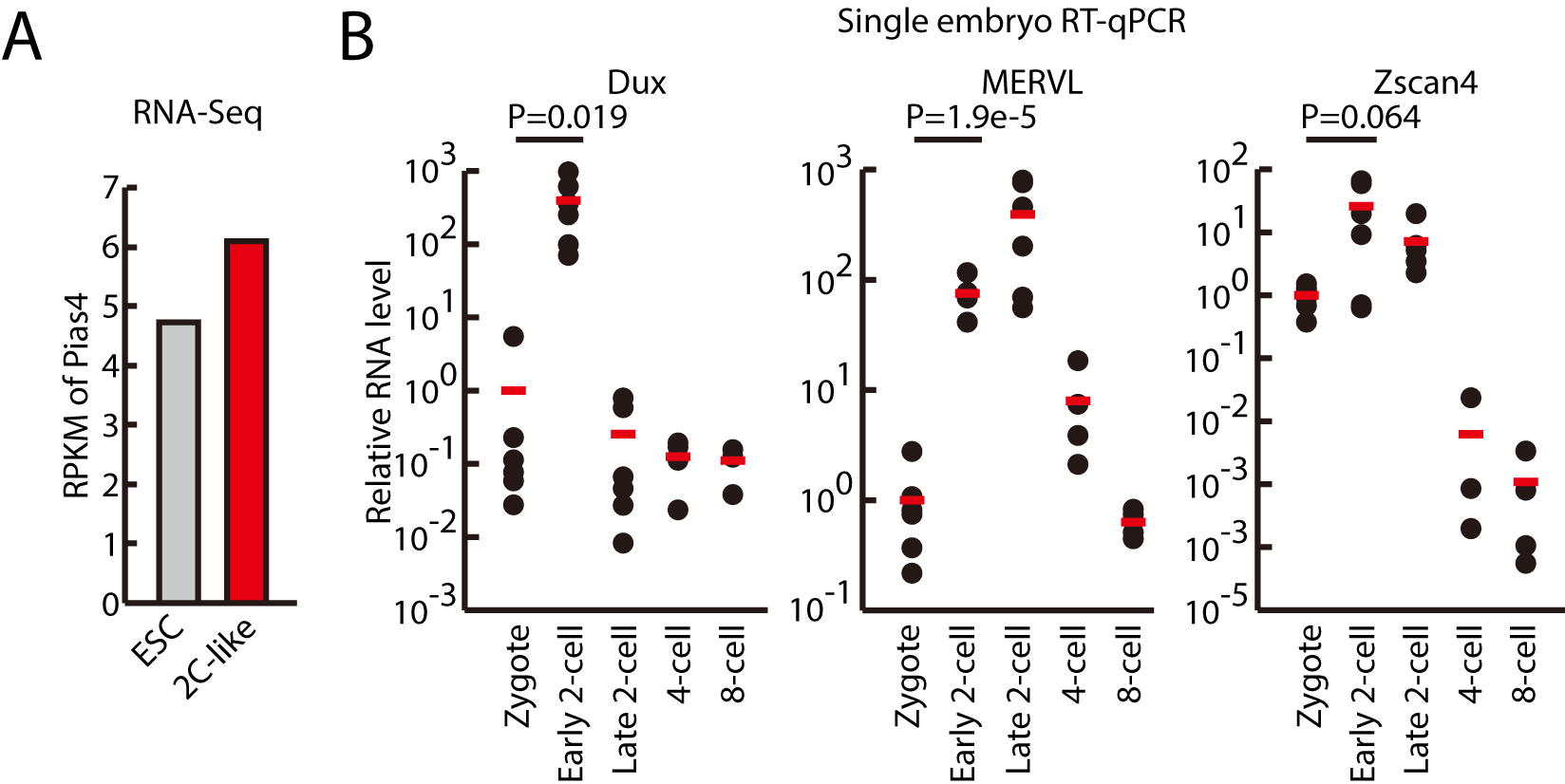

Supplement: S2 Fig — (A) Expression of Pias4 in 2C-like cells. RNA-seq data from [8]. (B) Single-embryo RT-qPCR of Dux, MERVL, and Zscan4d in preimplantation mouse embryos. Spike-in GFP mRNA was used as a control. Data were normalized to zygote. Red bars indicate mean. n = 4–6. Each dot represents one embryo. The p-value was as indicated, two-tailed Student’s t test. Source data for A and B can be found in the supplemental data file (S1 Data). Dux, double homeobox; GFP, green fluorescent protein; MERVL, murine endogenous retrovirus-L; Pias4, protein inhibitor of activated STAT 4; RNA-seq, RNA sequencing; RT-qPCR, quantitative reverse transcription PCR; Zscan4d, zinc figure and SCAN domain containing 4D. (TIF) [file pbio.3000324.s002.tif]

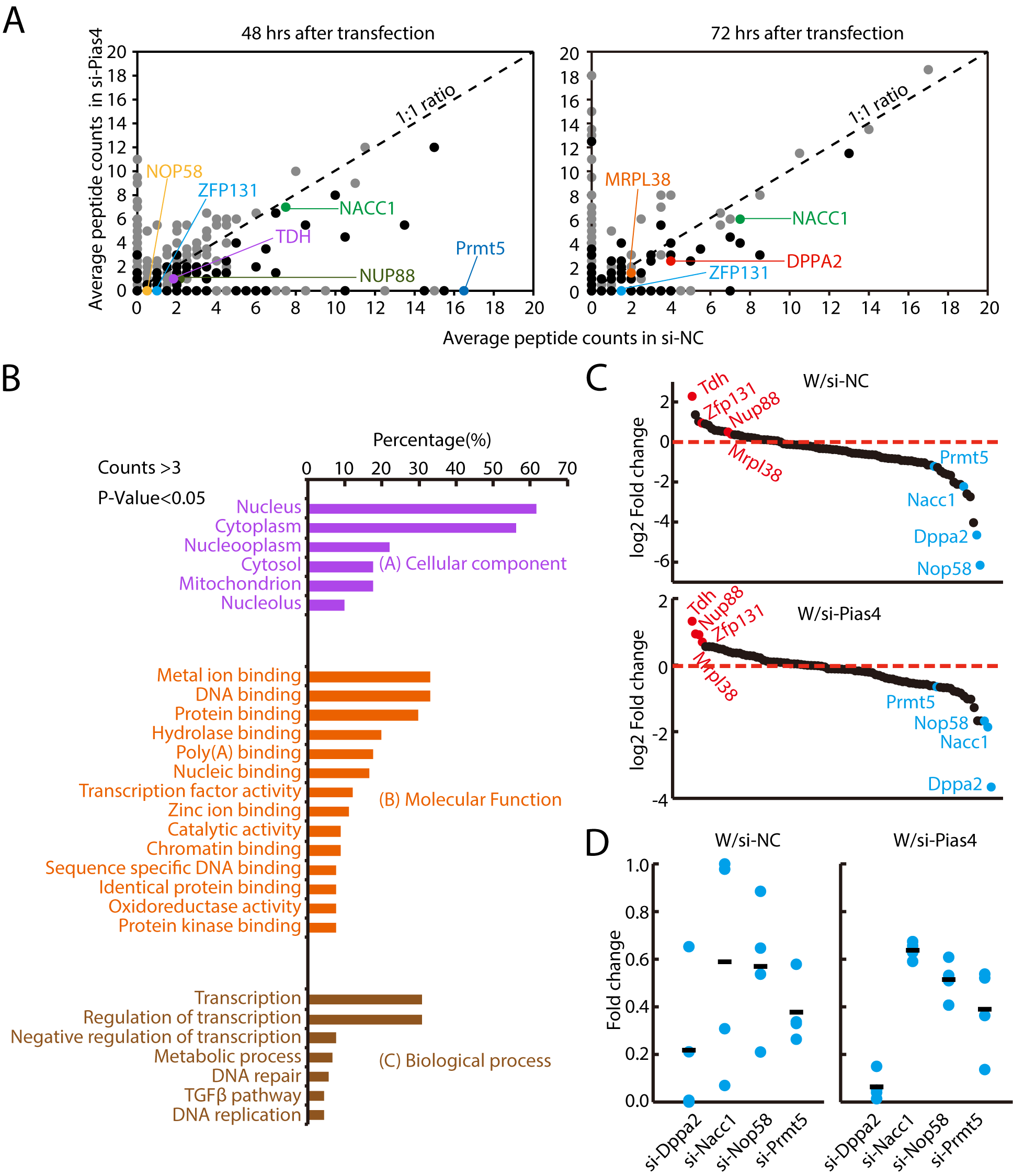

Supplement: S3 Fig — (A) Average peptide counts of proteins in 6xHis-Sumo2 pull-down/MS for ESCs treated with siRNAs against NC or Pias4. Proteins represented by black dots or colored dots were selected as candidate genes for CRISPRi screening. (B) Gene Ontology analysis of Pias4 substrates identified by Sumo2 IP. (C) Fold change of the percentage of Zscan4::GFP-positive cells in Pias4 substrate CRISPRi ESCs transfected with siNC or si-Pias4. Each dot represents an ESC line transfected with CRISPRi constructs targeting a candidate Pias4 substrate protein. The red line indicate the value 1.0. To calculate the fold change, the fraction of Zscan4::GFP-positive cells in each samples is divided by the fraction of Zscan4::GFP-positive cells in control CRISPRi ESCs. (D) Fold change of the percentage of 2C::tdTomato-positive cells in ESCs transfected with siRNAs against various Pias4 substrates in the presence of siNC or si-Pias4. Black bars indicate mean. n = 3–4. To calculate the fold change, the fraction of MERVL::tdTomato-positive cells in ESCs treated with different siRNAs is divided by the fraction of MERVL::tdTomato-positive cells ESCs treated with siNC. Source data for C and D can be found in the supplemental data file (S1 Data). CRISPRi, clustered regularly interspaced short palindromic repeat interference; ESC, embryonic stem cell; GFP, green fluorescent protein; IP, immunoprecipitation; MERVL, murine endogenous retrovirus-L; MS, mass spectrometry; NC, negative control; Pias4, protein inhibitor of activated STAT 4; siRNA, small interfering RNA; Sumo2, small ubiquitin-like modifier; tdTomato, tandem dimeric Tomato; Zscan4, zinc finger and SCAN domain containing 4. (TIF) [file pbio.3000324.s003.tif]

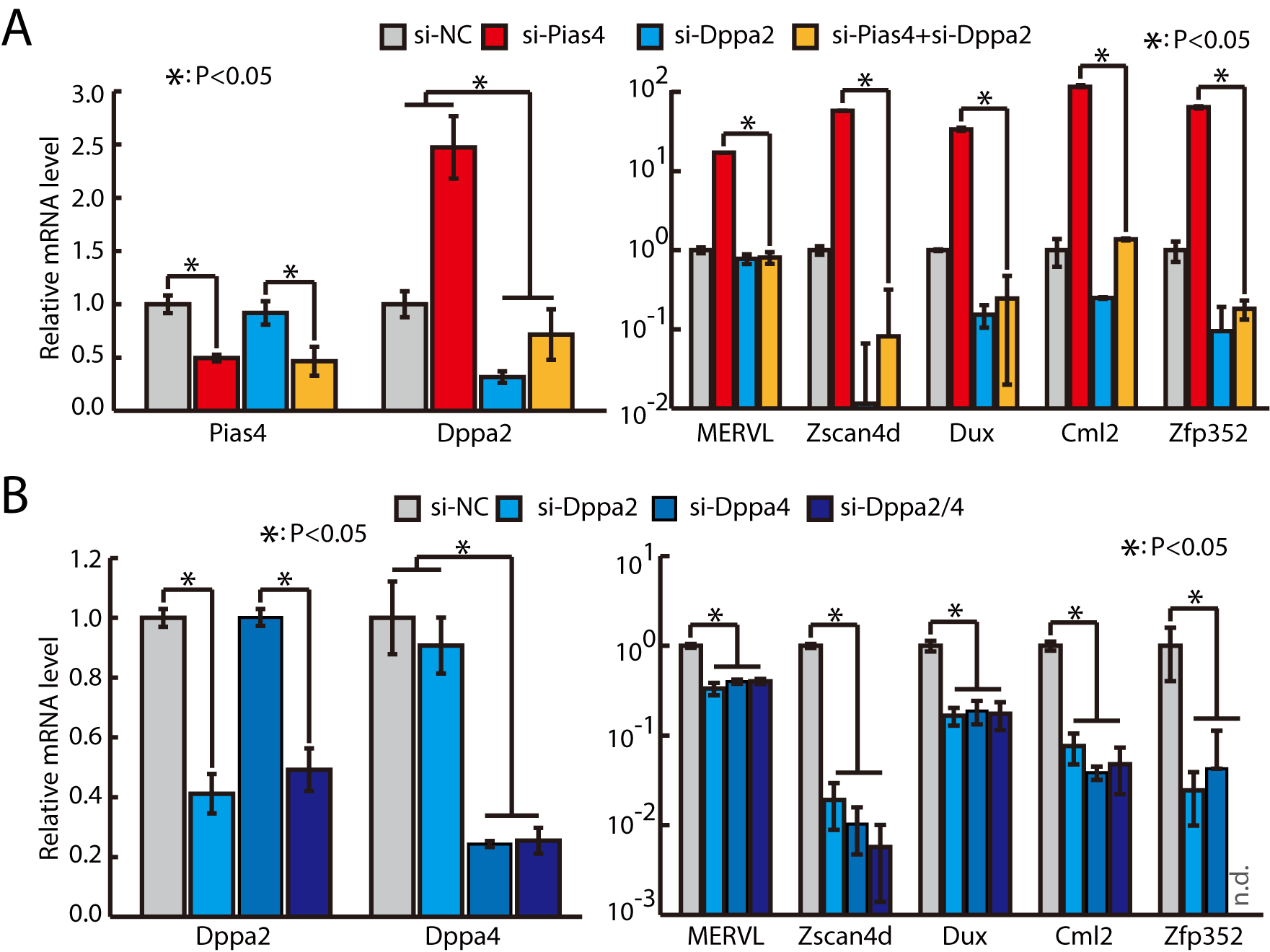

Supplement: S4 Fig — (A) RT-qPCR of Pias4 and Dppa2 (left), Dux, and other 2C-specific genes (right) in ESCs treated with siRNAs against Pias4 and Dppa2 individually or in combination. The β-actin gene was used as a control. For each gene, data were normalized to the mRNA level of wild-type ESCs. Shown are mean ± SD, n = 3. The p-value was calculated by two-way ANOVA followed by two-tailed Dunnett's test. (B) RT-qPCR of Dppa2 and Dppa4 (left), Dux, and other 2C-specific genes (right) in ESCs treated with siRNAs against Dppa2 and Dppa4 individually or in combination. The β-actin gene was used as a control. For each gene, data were normalized to the mRNA level of wild-type ESCs. Shown are mean ± SD, n = 3. The p-value was calculated by two-way ANOVA followed by two-tailed Dunnett's test. Source data for A and B can be found in the supplemental data file (S1 Data). 2C, 2-cell; Dppa, developmental pluripotency associated; Dux, double homeobox; ESC, embryonic stem cell; Pias4, protein inhibitor of activated STAT 4; RT-qPCR, quantitative reverse transcription PCR; siRNA, small interfering RNA. (TIF) [file pbio.3000324.s004.tif]

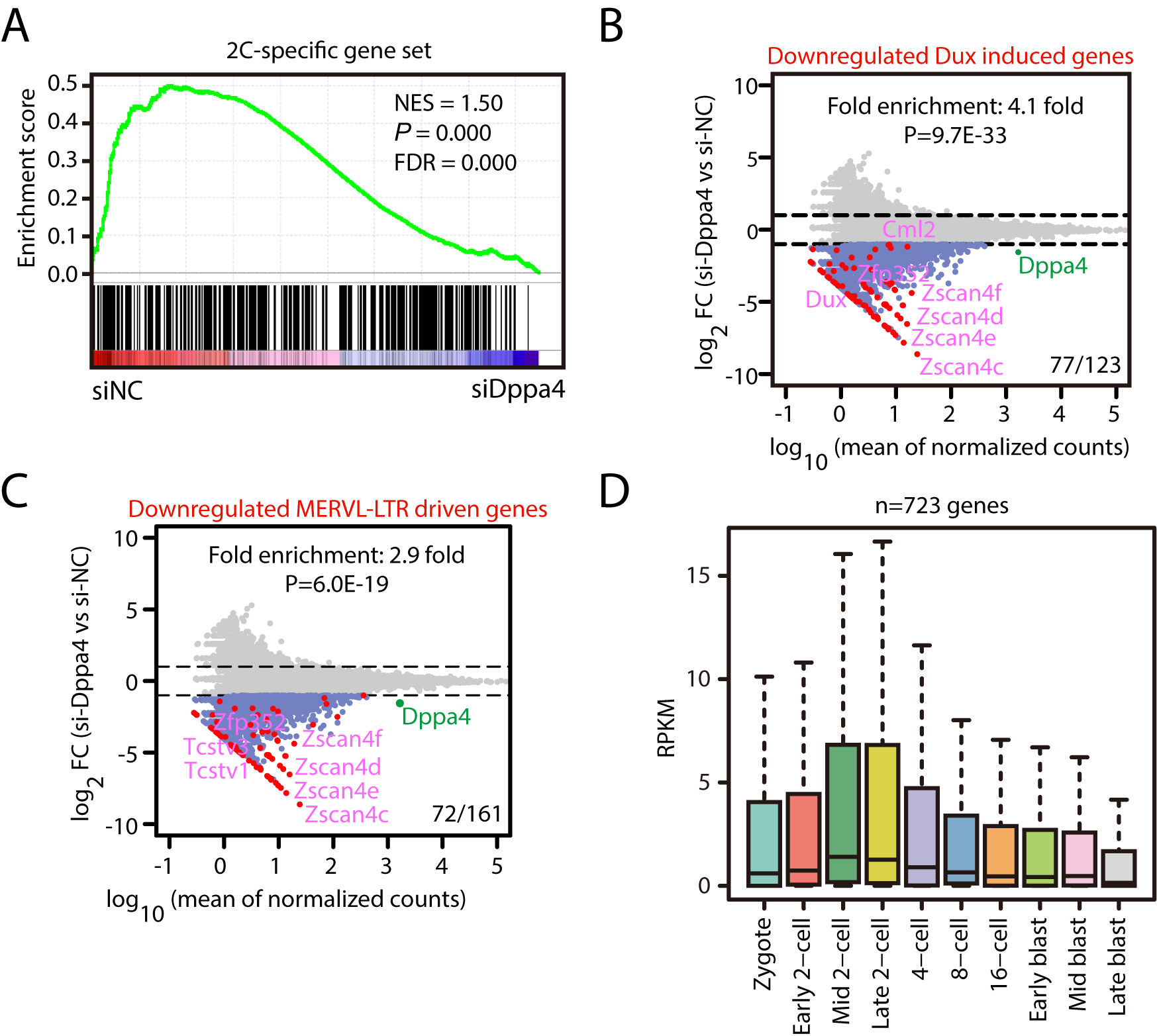

Supplement: S5 Fig — (A) GSEA for 2C-specific genes in ESCs transfected with control siRNAs or siRNAs against Dppa4. For the x-axis, genes were ranked based on the ratio of siNC versus si-Dppa2 ESCs. (B) MA plots showing gene expression changes in Dppa4-knockdown ESCs. Red dots indicate Dux-induced genes. Out of 123 Dux-induced genes, 77 were down-regulated in Dppa4-knockdown ESCs. Fold enrichment and p-value are shown. The p-value was calculated by hypergeometric test. (C) MA plots showing gene expression changes in Dppa4-knockdown ESCs. Red dots indicate MERVL–LTR-driven genes. Out of 161 MERVL–LTR-driven genes, 72 were down-regulated in Dppa4-knockdown ESCs. Fold enrichment and p-value are shown. The p-value was calculated by hypergeometric test. (D) Expression of genes down-regulated in Dppa4-knockdown ESCs in preimplantation mouse embryos. Center line, median; box limits, upper and lower quartiles; whiskers, 1.5× interquartile range. Preimplantation RNA-Seq data are from [32]. 2C, 2-cell; Dppa, developmental pluripotency associated; Dux, double homeobox; ESC, embryonic stem cell; GSEA, gene set enrichment analysis; LTR, long terminal repeat; MERVL, murine endogenous retrovirus-L; RNA-seq, RNA sequencing; siNC, siRNA against negative control; siRNA, small interfering RNA. (TIF) [file pbio.3000324.s005.tif]

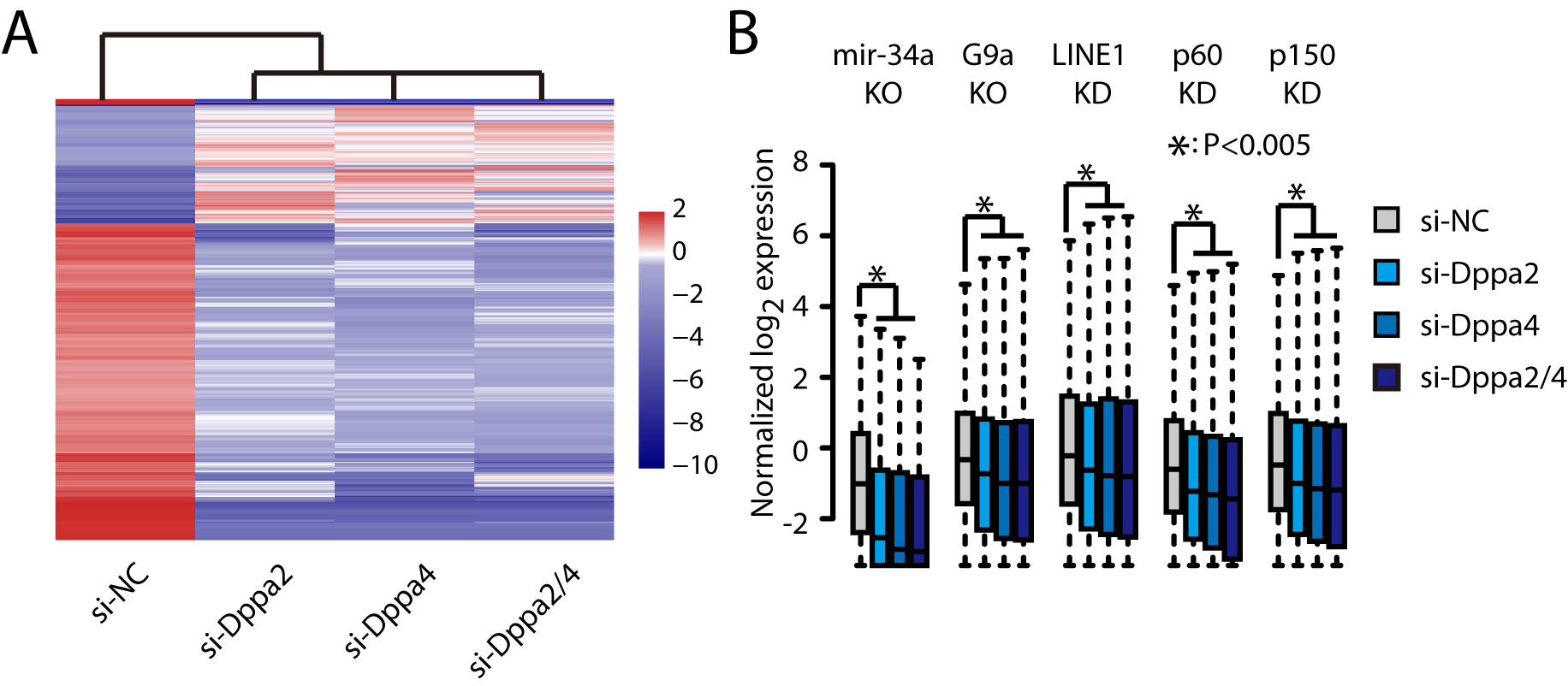

Supplement: S6 Fig — (A) Heatmap showing gene expression changes in ESCs transfected with control siRNAs and siRNAs against Dppa2 and Dppa4 individually or in combination. Only differentially expressed genes are shown. For each gene, data were normalized to the average of four samples. Color key is shown right (Log2). (B) Box-and-whisker plots showing expression of genes up-regulated by mir-34a knockout, G9a knockout, LINE1 knockdown, and Caf-1 p150 or p60 subunit knocked down in cells transfected with Dppa2 siRNAs or Dppa4 siRNAs separately or in combination. The p-value was determined by Wilcoxon signed rank test. Caf-1, chromatin assembly factor; Dppa, developmental pluripotency associated; ESC, embryonic stem cell; LINE1, long interspersed nuclear element; mir-34a, microRNA 34a; siRNA, small interfering RNA. (TIF) [file pbio.3000324.s006.tif]

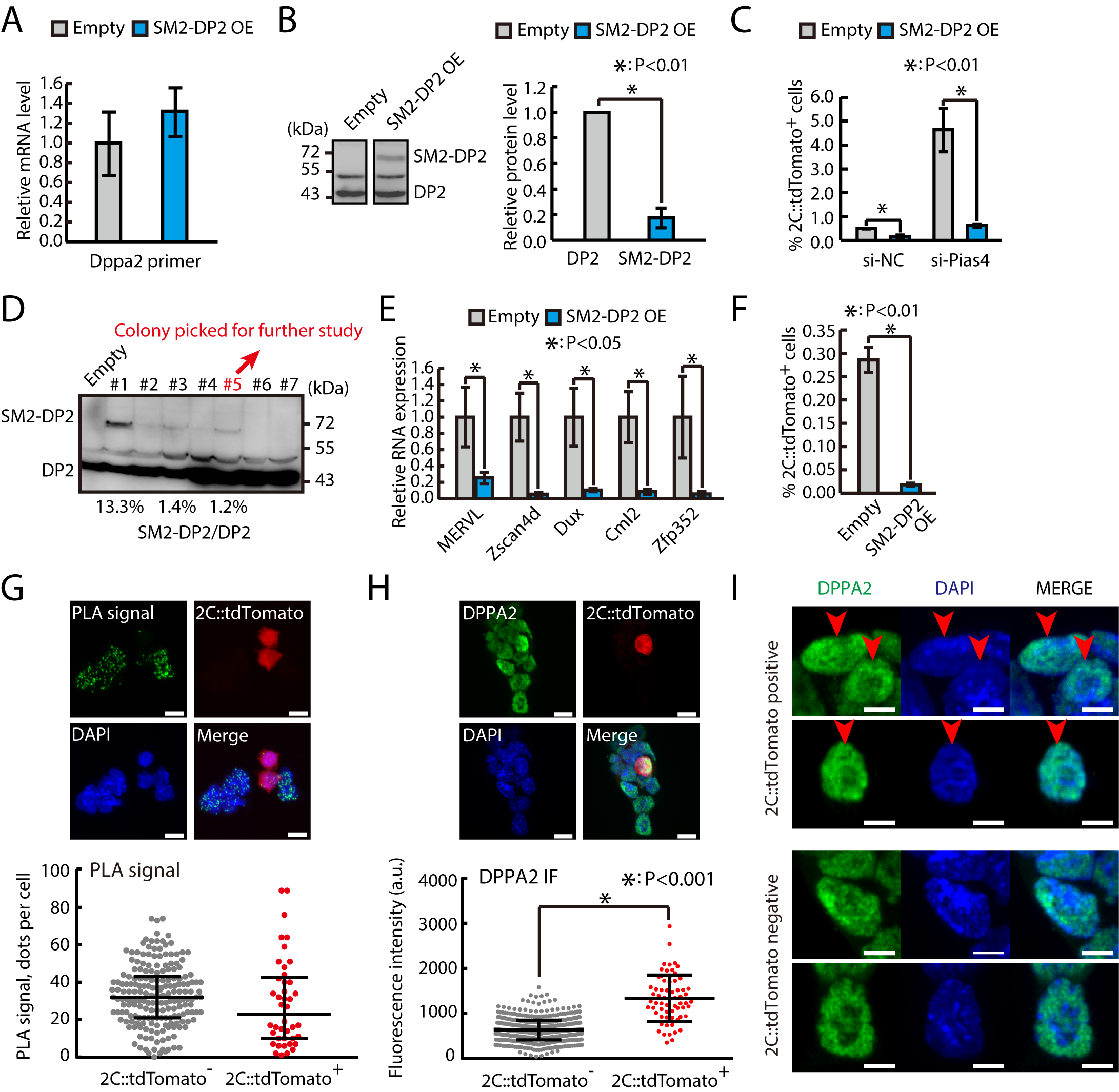

Supplement: S7 Fig — (A) RT-qPCR using Dppa2 primers in Sumo2ΔGG–Dppa2 overexpressing ESCs. The β-actin gene was used as a control. Data were normalized to the mRNA level of control ESCs. Shown are mean ± SD, n = 3. (B) Western blotting analysis of sumoylated and nonsumoylated Dppa2 in control and Sumo2ΔGG–Dppa2-overexpressing ESCs. This ESC colony was used for experiments in Fig 6B–6D. Left, representative gel blot images; right, relative ratio of Sumo2ΔGG–Dppa2 versus nonsumoylated Dppa2 in Sumo2ΔGG–Dppa2-overexpressing ESCs. Shown are mean ± SD, n = 3. The p-value was calculated by two-tailed Student's t test. (C) Flow cytometry analysis of control and Sumo2ΔGG–Dppa2-overexpressing ESCs treated with NC and Pias4 siRNAs. Shown are presented as mean ± SD, n = 3. The p-value was calculated by two-tailed Student’s t test. (D) Western blotting analysis of sumoylated and nonsumoylated Dppa2 in control and various Sumo2ΔGG–Dppa2-overexpressing ESCs. The ratio of sumoylated versus nonsumoylated Dppa2 is shown at the bottom of the gel. The colony #5 is picked for experiments in E and F. (E) RT-qPCR of Dux and other 2C-specific genes in control and #5 Sumo2ΔGG–Dppa2-overexpressing ESCs. The β-actin gene was used as a control. For each gene, data were normalized to the mRNA level of wild-type ESCs. Shown are mean ± SD, n = 3. The p-value was calculated by two-tailed Student’s t test. (F) Fraction of 2C::tdTomato-positive cells in control and #5 Sumo2ΔGG–Dppa2-overexpressing ESCs. Data are presented as mean ± SD, n = 3. The p-value was calculated by two-tailed Student’s t test. (G) PLA assay of SUMO2 and DPPA2 in 2C::tdTomato-positive and negative ESCs. Shown are representative images (left) and quantification of number of PLA dots/nucleus with mean ± SD (right). Scale bars, 10 µm. n = 42 tdTomato-positive cells and 194 tdTomato-negative cells. Each dot represents one nucleus. (H) IF staining of DPPA2 protein in MERVL::tdTomato-positive and negative ESCs. Shown are representative images (left) a [file pbio.3000324.s007.tif]

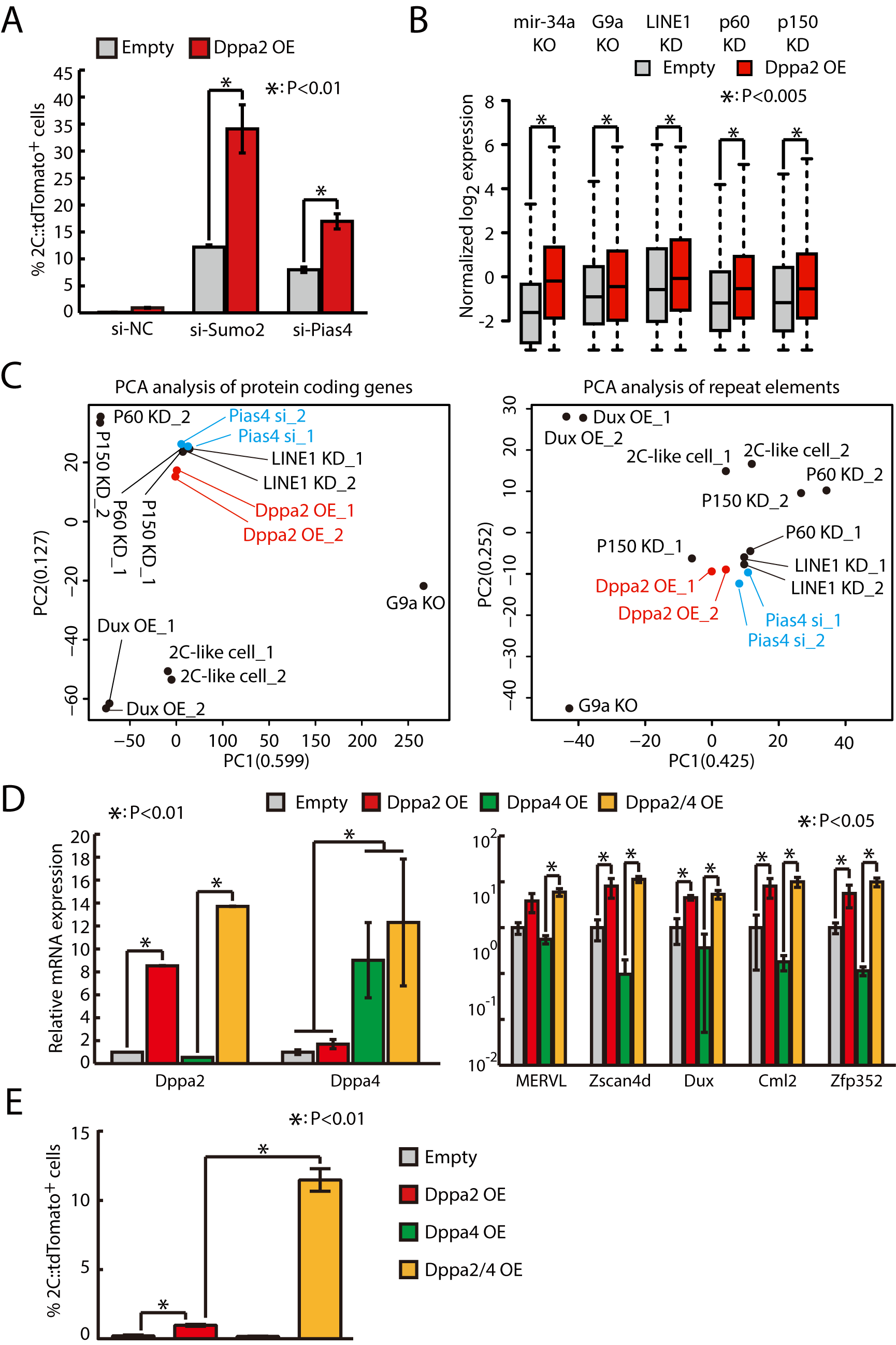

Supplement: S8 Fig — (A) Fraction of 2C::tdTomato-positive cells in control and Dppa2-overexpressing ESCs treated with Sumo2 or Pias4 siRNAs. Data are presented as mean ± SD, n = 3. The p-value was calculated by two-tailed Student’s t test. (B) Box-and-whisker plots showing expression of genes up-regulated by mir-34a KO, G9a KO, LINE1 knockdown, and Caf-1 p150 or p60 subunit knockdown in cells overexpressing Dppa2. The p-value was determined by Wilcoxon signed rank test. (C) PCA mapped scatter plot: global protein coding genes (left) and repeat elements (right). Data for G9a KO from [5], P150 and P60 knockdown and 2C::EGFP+ cells from [8], Dux-overexpressing cells from [11], and LINE1-knockdown cells from [14]. Data were normalized to the control cell line of each study to exclude batch effects before PCA processing. (D) RT-qPCR of Dux and other 2C-specific genes in control, Dppa2-, Dppa4-, or Dppa2/4-overexpressing ESCs. The β-actin gene was used as a control. For each gene, data were normalized to the mRNA level of control ESCs. Shown are mean ± SD, n = 3. The p-value was calculated by one-way ANOVA followed by two-tailed Dunnett's test. (E) Fraction of 2C::tdTomato-positive cells in control, Dppa2-, Dppa4-, or Dppa2/4-overexpressing ESCs. Data are presented as mean ± SD, n = 3. The p-value was calculated by two-tailed Student’s t test. Source data for A, D, and E can be found in the supplemental data file (S1 Data). 2C, 2-cell; Dppa, developmental pluripotency associated; Dux, double homeobox; EGFP, enhanced green fluorescent protein; ESC, embryonic stem cell; KO, knockout; LINE1, long interspersed nuclear element; mir-34a, microRNA 34a; PCA, principle component analysis; Pias4, protein inhibitor of activated STAT 4; RT-qPCR, quantitative reverse transcription PCR; siRNA, small interfering RNA; Sumo2, small ubiquitin-like modifier 2; tdTomato; tandem dimeric Tomato. (TIF) [file pbio.3000324.s008.tif]
